# Protein-Corona-by-Design in 2D: A Reliable Platform to Decode Bio–Nano Interactions for the Next-Generation Quality-by-Design Nanomedicines

**DOI:** 10.1002/adma.201802732

**Published:** 2018-08-24

**Authors:** Kuo-Ching Mei, Artur Ghazaryan, Er Zhen Teoh, Huw D. Summers, Yueting Li, Belén Ballesteros, Justyna Piasecka, Adam Walters, Robert C. Hider, Volker Mailänder, Khuloud T. Al-Jamal

**Affiliations:** Institute of Pharmaceutical Science, Faculty of Life Science & Medicine, King’s College London, Franklin-Wilkins Building, 150 Stamford Street, London SE1 9NH, UK; Max Planck Institute for Polymer Research, Ackermannweg 10, 55128 Mainz, Germany; Institute of Pharmaceutical Science, Faculty of Life Science & Medicine, King’s College London, Franklin-Wilkins Building, 150 Stamford Street, London SE1 9NH, UK; Centre for Nanohealth, Swansea University, Singleton Park, Swansea SA2 8PP, UK; Institute of Pharmaceutical Science, Faculty of Life Science & Medicine, King’s College London, Franklin-Wilkins Building, 150 Stamford Street, London SE1 9NH, UK; Key Laboratory of Pharmaceutics of Guizhou Province, Guizhou Medical University, No. 9, Beijing Road, Yunyan District, Guiyang 550004, China; Catalan Institute of Nanoscience and Nanotechnology (ICN2), CSIC and BIST, Campus UAB, Bellaterra, 08193 Barcelona, Spain; Centre for Nanohealth, Swansea University, Singleton Park, Swansea SA2 8PP, UK; Institute of Pharmaceutical Science, Faculty of Life Science & Medicine, King’s College London, Franklin-Wilkins Building, 150 Stamford Street, London SE1 9NH, UK; Institute of Pharmaceutical Science, Faculty of Life Science & Medicine, King’s College London, Franklin-Wilkins Building, 150 Stamford Street, London SE1 9NH, UK; Max Planck Institute for Polymer Research, Ackermannweg 10, 55128 Mainz, Germany; Department of Dermatology, University Medical Center Mainz, Johannes Gutenberg-University Langenbeckstr. 1, 55131 Mainz, Germany; Institute of Pharmaceutical Science, Faculty of Life Science & Medicine, King’s College London, Franklin-Wilkins Building, 150 Stamford Street, London SE1 9NH, UK

**Keywords:** click chemistry, drug delivery, graphene, nanomedicine, protein corona, toxicity

## Abstract

Hard corona (HC) protein, i.e., the environmental proteins of the biological medium that are bound to a nanosurface, is known to affect the biological fate of a nanomedicine. Due to the size, curvature, and specific surface area (SSA) 3-factor interactions inherited in the traditional 3D nanoparticle, HC-dependent bio–nano interactions are often poorly probed and interpreted. Here, the first HC-by-design case study in 2D is demonstrated that sequentially and linearly changes the HC quantity using functionalized graphene oxide (GO) nanosheets. The HC quantity and HC quality are analyzed using NanoDrop and label-free liquid chromatography–mass spectrometry (LC-MS) followed by principal component analysis (PCA). Cellular responses (uptake and cytotoxicity in J774 cell model) are compared using imaging cytometry and the modified lactate dehydrogenase assays, respectively. Cellular uptake linearly and solely correlates with HC quantity (*R*^2^ = 0.99634). The nanotoxicity, analyzed by retrospective design of experiment (DoE), is found to be dependent on the nanomaterial uptake (primary), HC composition (secondary), and nanomaterial exposure dose (tertiary). This unique 2D design eliminates the size–curvature–SSA multifactor interactions and can serve as a reliable screening platform to uncover HC-dependent bio–nano interactions to enable the next-generation quality-by-design (QbD) nanomedicines for better clinical translation.

Hard corona (HC) protein, i.e., proteins that are strongly associated with the surface of nanomaterials in biological environments, has drawn much attention in the field of nanomedicine.[1–3] HC profiles critically affect the nanomedicine stealth effect,[3] cellular uptake,[4] nanoparticle pathophysiology,[5] and mitigate nanomaterial-induced cytotoxicity.[6] Carefully designed nanomedicine could orchestrate HC profiles that facilitate blood–brain barrier (BBB) translocation in vivo without using BBB-target ligands.[7] Investigation of HC on 3D nanoparticles is influenced by the surface charge/surface chemistry and the 3-factor interactions between the size, specific surface area (SSA, the surface area/mass ratio), and the curvature, i.e., both curvature and SSA are size-dependent factors for HC formation.[8] 2D nanomaterials, i.e., nanomaterials where the third dimension is almost negligible (≈1 nm per layer), have much greater SSA when compared to 3D nanomaterials. For 2D nanomaterials, SSA is mass-dependent but not size-dependent; surface curvature is also not size- and SSA-dependent. It is, therefore, proposed as a better platform to clarify and uncover the HC-dependent bio–nano interactions. In this study, click-chemistry-modified 2D graphene oxide (GO) platform was used to examine the effect of NanoSAR (structure–activity relationship)[9] on HC and cytotoxicity, while avoiding the complex multigeometry interactions.[10] GO is being increasingly investigated in biomedical fields, e.g., drug delivery, bioimaging, tissue engineering, and biosensing.[11] It has been physically or chemically functionalized in numerous ways, e.g., click chemistry,[10] mechanochemistry,[12,13] mechano-click-chemistry,[14] and is proposed as a nanoenabled drug delivery system (NanoDDS) due to its large SSA when compared to traditional liposomal 3D nanocarriers.[15] From molecular dynamics simulation studies, it is known that proteins interact with GO via *π*–*π* stacking and hydrogen bonding (namely with aromatic tyrosine residuals and positively charged arginine and lysine residuals).[16] It is hypothesized that the introduction of hydrophobic functional groups, e.g., azide and alkyne to GO could create steric hindrance restricting the access of protein and water molecules to the oxygen derivatives on GO planar surface and edges, respectively. Sequential changes in GO surface properties were hypothesized to influence HC formation and can be used as a screening tool to further explore the complex nature of the HC for nanomedicine.

GO was synthesized and characterized using Mei’s modified Hummers method, and further derivatized into clickable GOs, i.e., GO–N_3_, GO≡, and Click^2^ GO (C^2^GO) as previously reported.[10,12] The introduction of azide and alkyne were made through the azide-mediated nucleophilic epoxide ring-opening reaction and Steglich esterification of the carboxylic groups, respectively (Scheme 1).[17]

The film-like structure was confirmed for all materials using scanning transmission electron microscopy (STEM) and transmission electron microscopy (TEM), regardless of the chemical functionalization (Figure S1, Supporting Information). GO, GO–N_3_, GO≡, and C^2^GO were washed (4×) with sterilized phosphate buffered saline (PBS) by centrifugation to afford washed pellets, which were resuspended and incubated with new-born calf serum (NCS) in vitro at 37 °C for 1 h to generate loosely bound and strongly bound soft corona (SC) and HC (see Figure S2 in the Supporting Information for workflow).[18]

SC and HC protein quantifications were performed using UV absorbance–based NanoDrop (Thermo Scientific) instead of bicinchoninic acid (BCA) assay due to the wider linear protein quantification ranges, better method reproducibility, and less day-to-day variability of the former (Figure S3A,B, Supporting Information). Overall protein recovery (total protein recovered from SC + HC + free post-incubation NCS) was 98+% for all tested samples, indicating a nearly complete HC desorption from the graphene, indirectly validating the use of this method for HC quantification (Figure S3C, Supporting Information). Variability in relative molar absorption coefficients was found to be less than 2% among the 4 HC samples (see the Supporting Information for additional details on calculations), so normalization step was not required for NanoDrop protein concentration readings (Figure S4, Supporting Information). As shown in Figure 1A, SC displayed an ascending range of protein quantities at 0.90 ± 0.05, 1.24 ± 0.06, 1.34 ± 0.11, and 1.52 ± 0.42 mg mg^−1^ for GO, GO–N_3_, GO≡, and C^2^GO, respectively. The sequential functionalization with azide and alkyne via blocking the planar- and edge oxygen-bearing functional groups increased the content of SC. HC quantity was found to be 1.44 ± 0.06, 0.92 ± 0.07, 1.06 ± 0.11, and 0.08 ± 0.38 mg mg^−1^ for GO, GO–N_3_, GO≡, and C^2^GO, respectively (Figure 1B). GO has the highest HC/SC ratio and the highest HC+SC quantity. Azide- and/or alkyne modifications dramatically decreased the HC quantity, but the former repels HC more efficiently. C^2^GO showed a synergistic azide-and-alkyne effect in repelling HC.

Quantitative label-free liquid chromatography–mass spectrometry (LC-MS) was then used to identify and quantify the HC composition as reported before.[3] NCS was used as the control serum. HCs from the GO, GO–N_3_, GO≡, and C^2^GO were detached from the graphene using a buffer solution at 95 °C for 5–10 min (see Table S1 in the Supporting Information for formulation) and analyzed in technical duplicates. The profiling results obtained by LC-MS were expressed as relative protein abundance (RPA) in percentage, as shown in Figure 2A. The top 30 proteins with more than 0.5% total protein RPA in at least one of the HC samples were selected for further analysis. The protein recovery from the top 30 proteins was 99+% for all HC samples. Other HC proteins found with <0.5% total protein RPA were neglected for further analysis. Each of the selected 30 HC proteins was ranked within each HC sample by the RPA (relative protein abundance among the 30 proteins), color-mapped and compared with the RPA of the control serum (see Table S2 in the Supporting Information for summarized protein information). Each graphene–HC RPA profile was regarded as 30 variables and analyzed using multivariate analysis (MVA), i.e., principal component analysis (PCA). PCA reduces the data into a smaller number of artificial variables known as the principal components (PC), that is, the linear combinations of the original variables (30 protein RPA per graphene type). The PC that accounts for (explained) the most, and the second most variances in the data (i.e., most of the data variances are happening along the PC coordinate) is known as the first PC (PC-1) and the second PC (PC-2), respectively.[19]

Two PC analyses were performed. First, RPA% of the control serum and the graphene–HCs was first analyzed to see if the serum RPA% is significantly different from the graphene–HC RPA%, i.e., to verify if graphene–HC RPA% is uniquely different from the serum. Second, the 4 graphene–HC RPA% were compared with each other to verify if the HC profile is significantly different. In brief, each HC profiling RPA% data can be considered as a vector in 30D space (each sample has 30 RPA measurements). Dimensionality reduction was performed by Eigenanalysis of the correlation matrix (where the variables are standardized; otherwise the proteins with small RPA would not contribute much to the analysis). The Eigenvalues (the fold changes/stretches of the Eigenvectors after linear transformation, i.e., the vector scalar) and the Eigenvectors of each PCs were obtained. Eigenvalues are shown in the PC screening plots (Figure S5A1, Supporting Information), to identify the number of the PCs needed to explain most of the variance in the data. The higher the PC Eigenvalue, the more variances are explained; for the first PCA analysis, since the PC-1a and PC-2a combined would cover +80% of the total Eigenvalues combined, the 30D vector can be dimensionally reduced into a 2D subspace while still capturing +80% of the variance in the data, i.e., the critical data information was maintained. The loading plot provides additional visual interpretation of the PC correlation by plotting the Eigenvectors of each variable for PC-1a and PC-2a (Figure 2B1 and Figure S5A2 (Supporting Information)). The PC-1a and PC-2a scores (shown in Figure 2B2) are the linear combinations of the original data. The larger the absolute value of the variable Eigenvector (either positive or negative), the greater importance of the corresponding variables (proteins) in calculating the PCs. Positive and negative Eigenvectors contribute to positive and negative PC scores, respectively. From Figure 2B2, a clear separation between serum-RPA profile and graphene-RPA profile based on PC-1a can be seen, i.e., there is a definite difference in the protein composition for the control serum and all the graphene–HC. Proteins with positive PC-1a Eigenvector values (24 out of 30 proteins tested) were found to have higher RPA on graphene derivatives, i.e., to have higher graphene binding affinity resulting in uniquely different graphene–HC profiles that are significantly different from the background serum protein profile (Figure 2B2, *x*-axis).

The second PCA for graphene–HC RPA% was analyzed similarly. The loading and screen plots are represented in Figure S5B (Supporting Information). The Eigenvectors of the PC-1b and PC-2b are shown in Figure 2C1. In the PCA score plot (Figure 2C2), all 4 graphene–HC are separated from each other, i.e., being significantly different. Positive PC-1b scores (attributed by proteins with positive PC-1b Eigenvectors preferentially bind GO–N_3_, GO≡, and C^2^GO) were found in GO–N_3_, GO≡, and C^2^GO, when compared to negatively scored GO (Figure 2C2, *x*-axis), i.e., the click chemistry–enabled GO displayed a different HC profile when compared to GO. In addition, the azide and alkyne double functionalized C^2^GO was well-separated from the monofunctionalized GO–N_3_ and GO≡ by PC-2b scores.

To translate PCA results from artificial PC units (PC scores) into more readily interpretable biological functions, and to verify how PC scores are related to bio–nano interactions, correlation studies were performed. The first correlation study was quantitative cellular uptake using J774 cells (mouse monocyte macrophage) incubated with all four GO derivatives at 20 μg mL^−1^ concentration for 24 h (see “Methods” and Figure S6 in the Supporting Information for methods and sample preparation). Cellular uptake was evaluated with an imaging cytometer,[20] which imaged cells individually before and after graphene treatments (Figure 3A). Changes in the cell image bright-field light intensity (BFI) were normalized to the cell area. The lower the BFI, the higher the GO uptake, i.e., J774 uptake: C^2^GO > GO–N_3_ > GO≡ > GO (Figure 3B). The BFI of untreated J774 cells was then subtracted from the graphene groups; the resulting net BFI changes and HC/graphene w/w values were plotted as a function of the PC-1b scores and PC-2b scores to obtain correlation plots (Figure 3C,D). PC1b scores were found to be linearly correlated with both HC/graphene and BFI with *R*^2^ = 0.99626 and 0.98536, respectively; no correlation was found for PC-2b scores. A direct linear relationship (*R*^2^ = 0.99634) was also found between the HC quantity and the changes in the BFI. The higher the HC quantity, the lower the cellular uptake, and the PC-1b score, i.e., the PC-1b score accurately represented, and can be translated into the HC quantity and cellular uptake level.

As the PC-2b score is not related to HC quantity and cellular uptake, the translation of PC-2b scores continued and hypothesized to be associated with another critical aspect of bio–nano interactions: cytotoxicity/cell viability. A correlation study between the PCs and cell viability was performed by treating J774 cells with GO derivatives at 10, 50, 100 μg mL^−1^ for 72 h (37 °C, 5% CO_2_). The 72 h cell viability results showed significant dose-dependent toxicity for all four GO derivatives (Figure 4A). The material-dependent cell viability was found in the following order: GO > GO–N_3_ > GO≡ > C^2^GO. Superoxide dismutase (SOD) activity assay was performed for the viable cells after graphene treatment to verify if the cytotoxicity is related to oxidative stress. The SOD activity in viable J774 cells decreased sequentially similar to the viability trend, i.e., GO > GO–N_3_ > GO≡ > C^2^GO, implying an increased oxidative stress from GO to C^2^GO (Figure S7, Supporting Information). However, the SOD assay is not an ideal indicator for modeling bio–nano interactions due to the inherent data selection bias, i.e., only viable cells are analyzed. Lactate dehydrogenase (LDH) assay, which analyzes cell viability by taking into account the cell population as a whole (living and dead cells), was selected for further correlation analysis.

The cell viability trend, i.e., C^2^GO < GO≡ < GO–N_3_ < GO, is partially different from the order found in cellular uptake studies: C^2^GO > GO–N_3_ > GO≡ > GO. GO–N_3_ was taken up more than GO≡ but appeared to be less toxic, i.e., the cellular uptake/HC quantity (PC-1b scores) does not translate directly into cytotoxicity results, other factors were involved. Linear regressions were performed between the PC-1b scores, cell viabilities, and relative SOD activities across three different dose ranges (10, 50, and 100 μg mL^−1^). The resulting *R*^2^ ranged between 0.87 and 0.93 (viability%, Figure 4B1), i.e., for overall cell viability, only ≈87–93 of the variances were explained by the PC-1b scores, respectively. PC-1b scores are particularly poor in describing the cell viability (*R*^2^ < 0.45) specifically for cells treated with click-modified graphene (GO–N_3_, GO≡, C^2^GO, Figure 4B2). A much better correlation (linearity) was found when cell viability (*R*^2^ = 0.96–0.99) (Figure 4B3) was plotted as functions of PC-2b scores across all dosing ranges. PC-2b scores could explain +96% of the variance seen among the click-modified derivatives, suggesting that PC-2b scores are well translated into cytotoxicity.

To better understand and visualize how overall cell viability is influenced by HC quantity/quality-dependent and dose-dependent factors (factor A: PC-1b scores; factor B: PC-2b scores, factor C: graphene dose in μg mL^−1^), a retrospective analysis using historical cell viability data (Figure 4A) was performed.[21] Response surfaces and the predictive equations were then established. Raw data used to create the predictive response surface (cell viability%) are summarized in Table S3 (Supporting Information). Data were analyzed using Design-Expert 9, v9.0.6.2 (Stat-ease, Inc., USA). A suitable predictive model was achieved using sequential model sum of squares (SMSS, Tables S4–S8, Supporting Information). A detailed description of the retrospective design of experiment (DoE) analysis is provided in the Supporting Information. The final fitted equation in terms of coded factors (so that factors of different units can be compared) is listed as the following

(1) $${\text{log}}_{10}(\text{Viability}\%)=1.30-0.36A+0.23B-0.18C$$

From Equation (1), cell viability was a collective consequence of all three factors, where the factor *A* (PC-1b score) has the largest effect (largest absolute coefficient = 0.36) followed by factor *B* (PC-2b score, absolute coefficient = 0.23; (0.23/0.36) × 100% = 64%, i.e., the influence of factor *B* is ≈64% of factor *A*), and factor *C* (graphene dose, absolute coefficient = 0.18; (0.18/0.36) × 100% = 50%, i.e., factor *C* is half as influential as factor *A*). These results suggest that the overall cell viability was mainly correlated with cellular uptake/HC quantity (low uptake GO vs high uptake C^2^GO). At higher uptake levels, e.g., GO–N_3_ versus GO≡ versus C^2^GO, proteins that orchestrated the HC (HC quality) are the dominating factors for cytotoxicity, despite questions remained for the exact mechanism of intracellular HC-induced cytotoxicity. Finally, the overall cytotoxicity is also dose-dependent with a lesser influence when compared to PC-1b and PC-2b scores (as reflected by the coefficient values of coded factors). The final fitted equations and response surface in terms of actual factor (actor factor units) is shown in Equation (2) and Figure 4C.

(2) $${\text{log}}_{10}(\text{Viability}\%)=1.42-0.08\times \text{PC}1\text{b}+0.06\times \text{PC}2\text{b}-0.004\times \text{Dose}$$

The more negative the PC-2b score is, the less viable the treated cells are (Figure 4B3,C). As the PC-2b score is contributed by all 30 proteins, negative scores are collectively summed by HC proteins with negative Eigenvectors. HC proteins with a larger negative PC-2b Eigenvector (e.g., vitamin D binding protein (DBP), intra-alpha-trypsin inhibitor heavy chain H2, beta-2 glycoprotein 1, and POTE Ankyrin domain family member F, Figure 2C1) are correlated with lower cell viability and higher oxidative stress. These proteins were more abundant in HC of C^2^GO (the most toxic derivative in vitro), except POTE Ankyrin domain family member F, where the RPA in GO > C^2^GO > GO–N_3_ > GO≡. The high RPA of POTE Ankyrin domain family member F in GO also explained the overall negative PC-2b score for GO. Proteins with larger positive PC-2b Eigenvectors (e.g., hemoglobin beta/delta, prothrombin, antithrombin-III, complement C9, Apo A-I, Apo C-III, gelsolin, and lactotransferrin) contributed more to positive PC-2b scores and translated to higher cell viability and lower oxidative stress. GO–N_3_ which demonstrated the highest biocompatibility among the derivatives also showed the highest RPA for hemoglobin beta/delta, Apo C-III, complement C9, prothrombin, antithrombin-III, and gelsolin, while having the lowest RPA for DBP.

Designing the HC in 2D provides a simplified platform allowing NanoSAR studies with fewer interferences. As hypothesized, the surface modifications blocked proteins binding presumably by steric hindrance as implied by the sequentially changing SC and HC, respectively (Figure 1A,B). Despite many studies attempted to modify 3D nanoparticles by decorating nanoparticles with different polymers and different chain lengths, only categorical HC-quality changes (e.g., preferentially absorb or repel certain HC proteins), material-based sequential HC-quantity changes (i.e., the same base material with various levels of HC quantity) were difficult to achieve.[3,22] There are previously reported HC studies on 2D graphene, which the change in HC quantity was achieved by changing the serum content in cell culture media with little control over the 2D surface;[6] such approach introduced additional systematic errors and led to difficulties to interpret NanoSAR findings.[23] Furthermore, the mitigation effect was mostly concluded for GO by qualitative TEM ultrastructural studies without further quantitative analysis.[6] To the best of our knowledge, this is the first study demonstrating the sequential changes of HC quantity and quality for potential NanoDDS, which also clarified the NanoSAR by quantitative NanoDrop–LC-MS/PCA (Figures 1 and 2). Correlation studies were performed to decode PC-1b and PC-2b scores, which were translated into HC quantity/cellular uptake (linearity *R*^2^ > 0.98, Figure 3), and cytotoxicity (modified lactate dehydrogenase assay activity linearity *R*^2^ > 0.96, Figure 4B3), respectively. Cellular uptake is almost completely HC-quantity-dependent. Cytotoxicity, however, is collectively affected by HC quantity, HC quality, and graphene exposure dose (Equations (1) and (2)), Figure 4C). For example, GO scored similarly to that of GO≡ in PC-2b, which should have translated into similar cytotoxicity. However, GO≡ is significantly more toxic to cells due to a much higher cellular uptake.

Intrinsic sequence-based protein properties, e.g., molecular weight, intrinsic solubility, and isoelectric charges,[24] were used in attempting to correlate protein properties with PCA scores (and biological translations) with no success (Table S2 and Figures S8–S14, Supporting Information). Structure-based protein PCA could be involved in future studies as protein functions are better described or predicted by sequence motif–based or structure-based methods, as demonstrated in other studies.[25] Questions remained to the mechanistic understanding of why any given HC protein is biocompatible or toxic, as the intracellular fate of the nanomaterial and the associated HC could also be different.[1] The usefulness of the 2D GO high-throughput platforms provides an easy way to screen multiple serum proteins for future single-(HC)-protein NanoSAR studies.

The first protein corona-by-design example in 2D was demonstrated to investigate the effects of HC-dependent bio–nano interactions. The click-modified 2D GO platform successfully creates material-dependent sequential changes in both HC quantity and HC quality while eliminating the size–curvature–SSA multifactor interactions. HC quantity influenced the degree of cellular uptake; while HC quantity (primary), HC quality (secondary), and material exposure dose (tertiary) influenced the cytotoxicity collectively. This study provides a 2D protein corona quality-by-design (QbD) platform with an accurate prediction of bio–nano interaction. Identifying “safe” and “toxic” protein corona profiles in 2D could pave the way for future QbD 2D/3D nanomedicines and better clinical translation.

## Supplementary Material

Supporting Information is available from the Wiley Online Library or from the author.

Supporting Information for Adv. Mater., DOI: 10.1002/adma.201802732

## Figures and Tables

**Figure 1:**
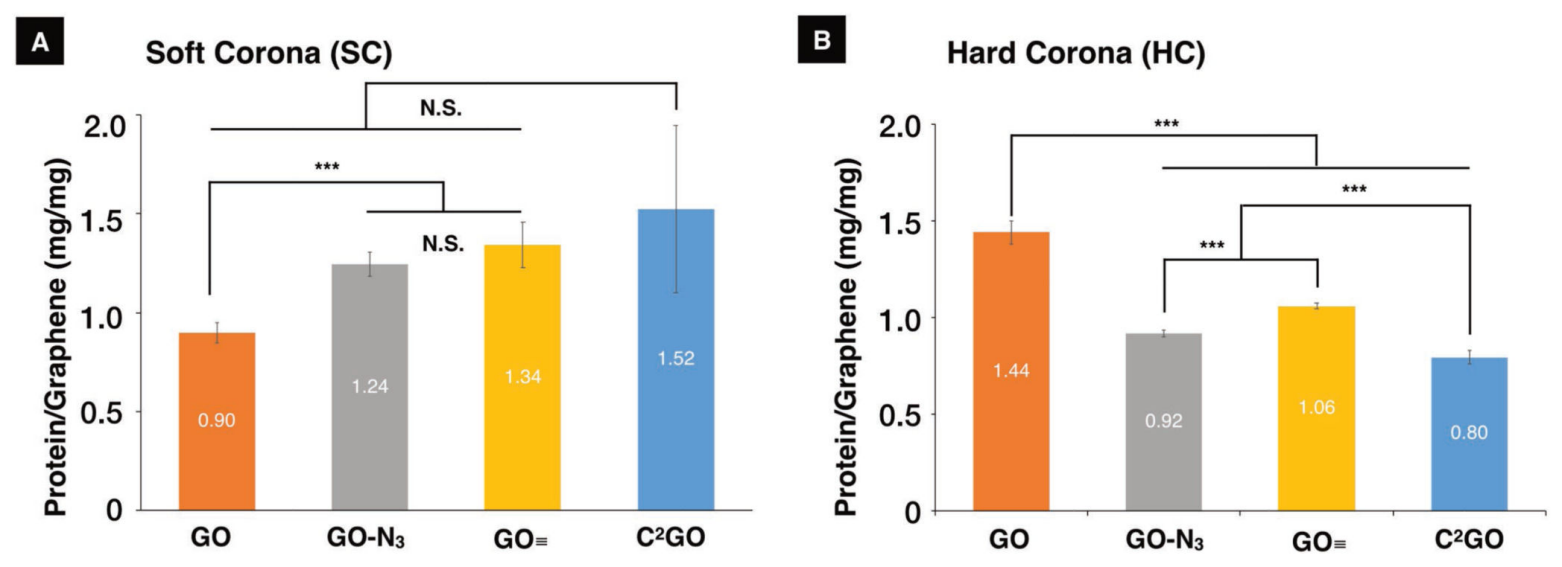
Protein corona analysis for GO, GO–N_3_, GO≡, and C^2^GO. A) An ascending SC quantities per mg of graphene materials was found to be 0.90 ± 0.05, 1.24 ± 0.06, 1.34 ± 0.11, and 1.52 ± 0.42 mg mg^−1^ for GO, GO–N_3_, GO≡, and C^2^GO, respectively (Levene’s test *p* = 0.014, Welch’s *F*(3, 6.09) = 30.09, *p* < 0.001. Post hoc = Games–Howell, adjusted *p* = N.S. > 0.05, * < 0.05, ** <0.01, *** < 0.001). B) A declining HC quantity per mg of graphene materials, obtained by direct measurements of proteins detached by sodium dodecyl sulfate (SDS) was found to be 1.44 ± 0.06, 0.92 ± 0.07, 1.06 ± 0.11, and 0.80 ± 0.38 mg mg^−1^ for GO, GO–N_3_, GO≡, and C^2^GO, respectively (Levene’s test *p* = 0.637. *F*(3, 8) = 174, *p* < 0.001. Post hoc = Tukey, adjusted *p*-value = N.S. > 0.05, * < 0.05, ** < 0.01, *** < 0.001).

**Figure 2:**
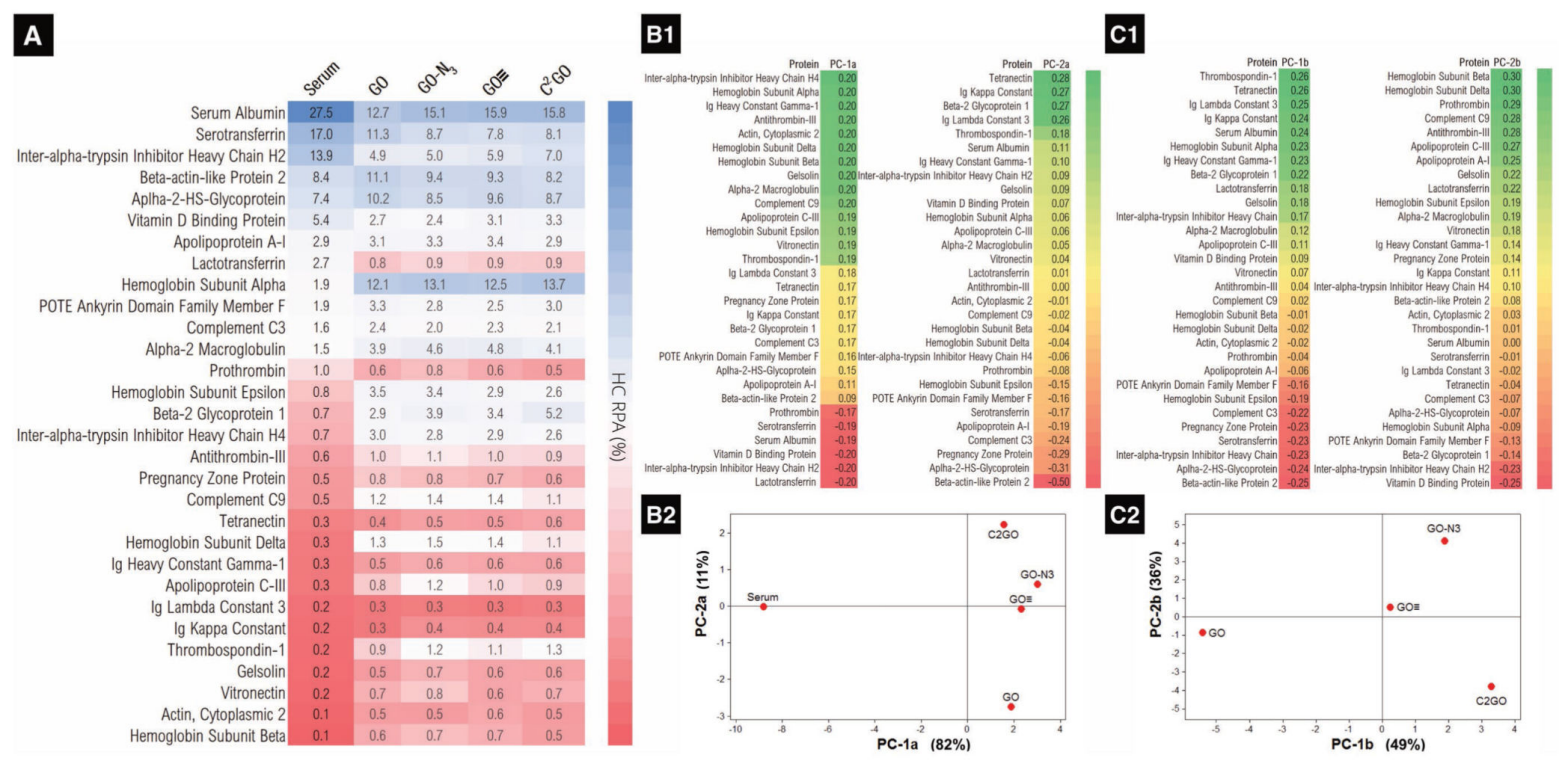
Relative protein abundance (RPA) profiling by LC-MS. A) The hard corona compositions analyzed by LC-MS were expressed as relative hard corona protein abundance in percentages for the individual groups. Each protein was ranked by the RPA and color-mapped for the serum, HC–GO, HC–GO–N_3_, HC–GO≡, and HC–C^2^GO. B) Principal component analysis (PCA) for RPA of serum and graphene RPA. The RPA values in (A) were analyzed by PCA. B1) The Eigenvectors of the PC-1a and PC-2a. B2) PCA score plot shows that the graphene RPA is separated from the serum RPA by the principal component 1 (PC-1a), i.e., graphene RPA is different from the control serum. The graphene RPAs are separated by PC-2a. C). PCA for graphene RPA. C1) The Eigenvectors of the PC-1b and PC-2b. C2) PCA score plot of the graphene RPA shows separation between the clickable graphene (GO–N_3_, GO≡, and C_2_GO) and GO by PC-1b scores. A separation between the monoclickable derivatives (GO–N_3_, GO≡) and the double-clickable derivative (C^2^GO) is seen by PC-2b scores.

**Figure 3:**
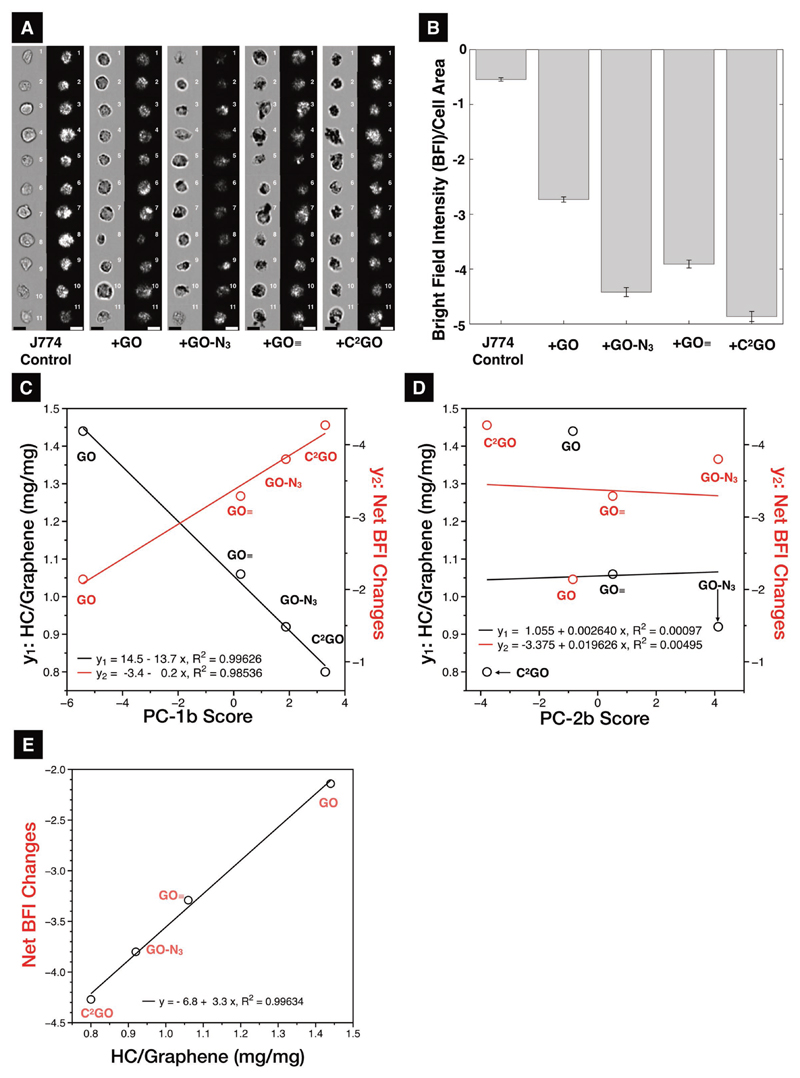
Cellular uptake assessments. A) Cellular uptake of the graphene derivatives in J774 cells analyzed by imaging cytometer. Representative images of the J774 cells ± graphene imaged from the bright-field and dark-field channels are shown (scale bar = 20 μm). B) The cellular uptake results were expressed as bright-field (light) intensity per cell area with darker cells having reduced bright-field intensity indicating higher uptake. C) Correlation analysis for the bright-field intensity/cell area, and function of HC quantity as functions of PC-1b scores. Linear relationships were found between PC-1b scores and HC quantity, and between PC-1b scores and cellular uptake, with a *R*^2^ = 0.99626 and 0.98536, respectively. The lowest HC quantity was found to result in the highest cellular uptake. D) Correlation analysis for the bright-field intensity/cell area, and HC quantity as functions of PC-2b scores. No meaningful correlation was found. E) Correlation analysis for the bright-field intensity/cell area as a function of HC quantity found a linear relationship with a *R*^2^ = 0.99634.

**Figure 4:**
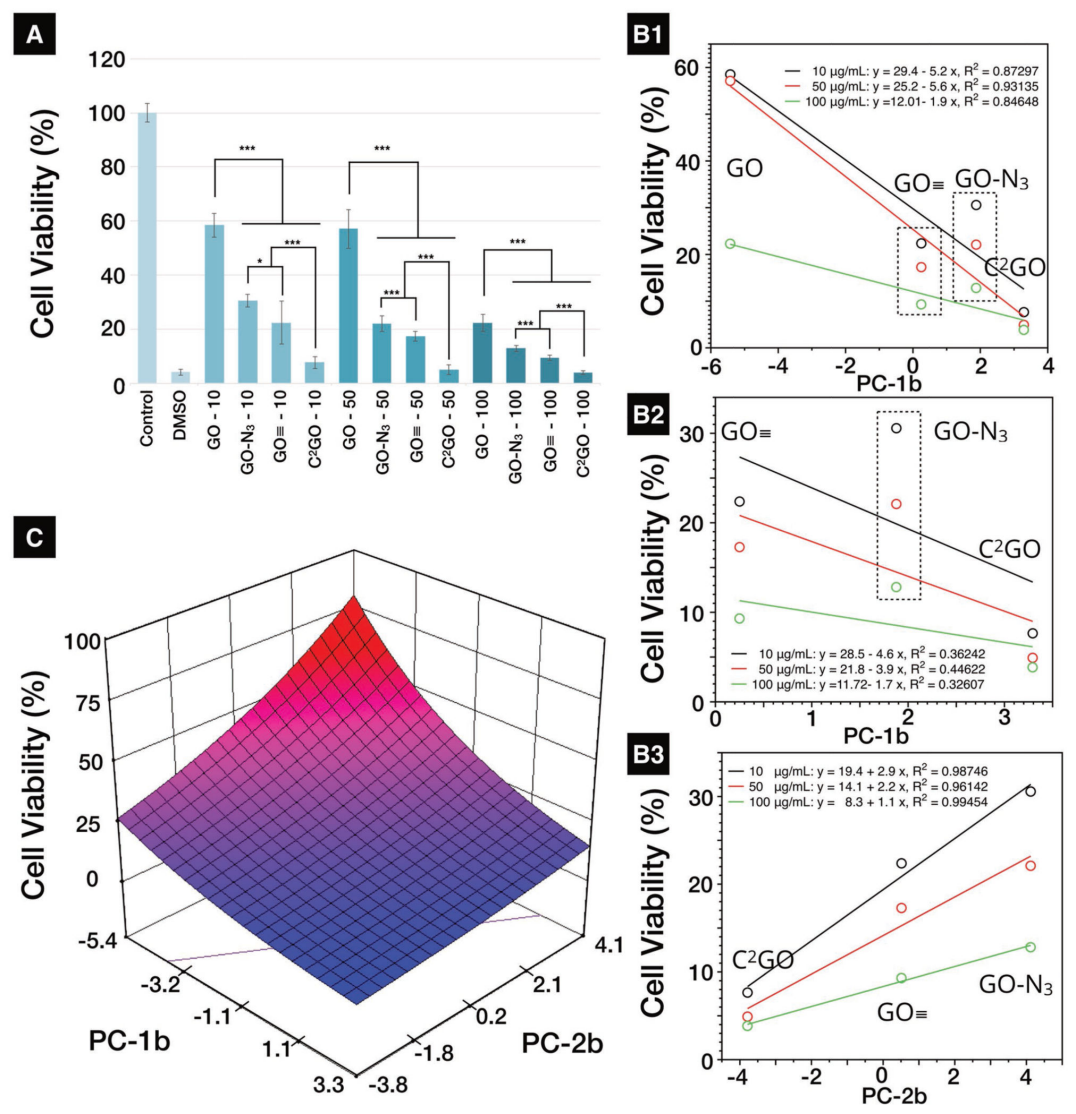
Cytotoxicity assessments. A) The modified lactate dehydrogenase (mLDH) assay. mLDH was used to evaluate the cell viability of GO, GO, GO–N_3_, GO≡, and C^2^GO at 10/50/100 μg mL^−1^ after 72 h incubation (37 °C, 5% CO_2_). In J774 cells (mouse monocyte macrophage cells), significant dose- and material-dependent toxicities were found in J774 (for 10 μg mL^−1^: Welch’s *F*(5, 30.8461) = 2788.81, *p* < 0.001; 50 μg mL^−1^: Welch’s *F*(5, 31.9966) = 2608.28; 100 μg mL^−1^: Welch’s *F*(5, 34.4422) = 2657.15, *p* < 0.001. Post hoc = Games–Howell, adjusted *p* = N.S. > 0.05, * < 0.05, ** < 0.01, *** < 0.001). B1–B3) Linear correlation studies between PC-1b score, PC-2b scores, and cell viability (%). C) Response surface of cell viability (dose = 55 μg mL^−1^, 72 h) as a function of both PC-1b and PC-2b scores.

**Scheme 1:**
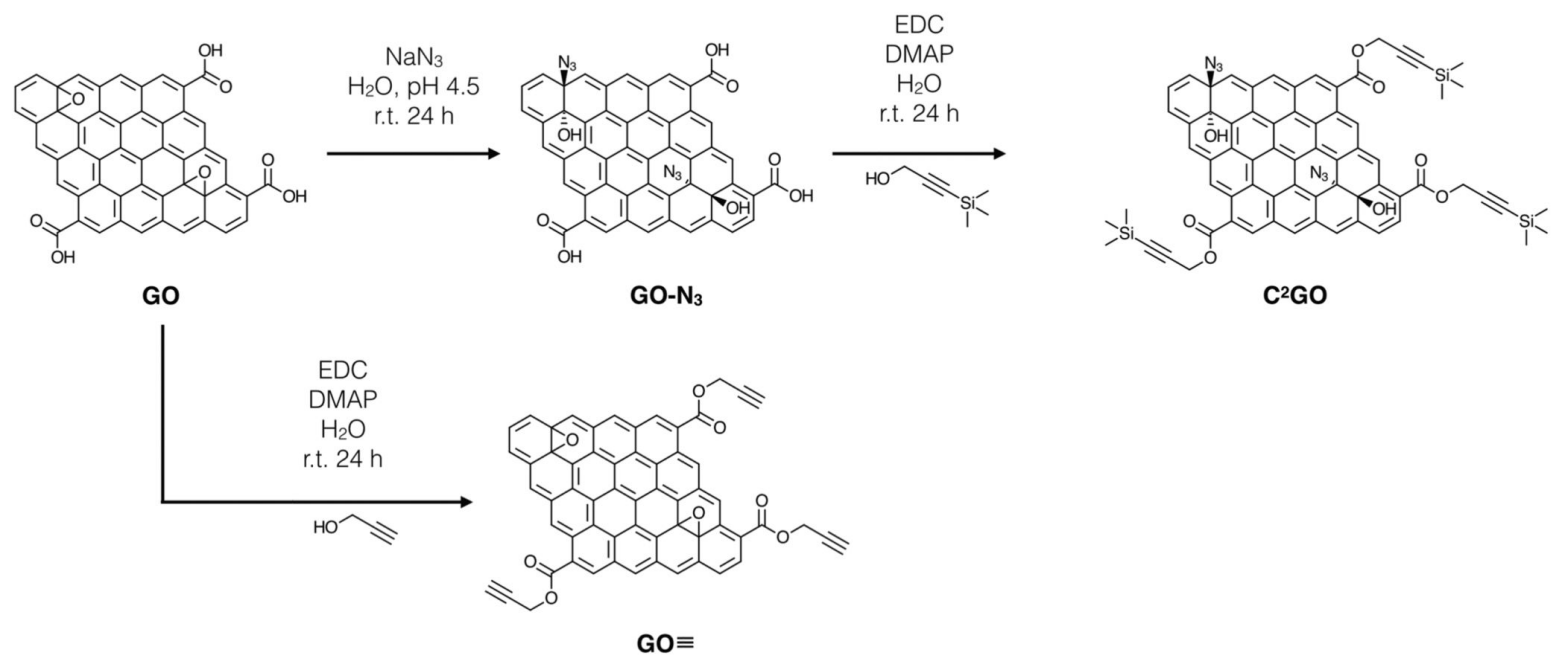
GO surface chemistry modification with azide and alkyne.
