# Supplementary material for: Protein-Corona-by-Design in 2D: A Reliable Platform to Decode Bio–Nano Interactions for the Next-Generation Quality-by-Design Nanomedicines
Source: Adv Mater. Author manuscript; Available in PMC 2020 Jan 9. (PMC6952277; doi:10.1002/adma.201802732)
Supplement: Supporting Information for Adv. Mater., DOI: 10.1002/adma.201802732 [file EMS82865-supplement-Supporting_Information.pdf]

Copyright WILEY-VCH Verlag GmbH & Co. KGaA, 69469 Weinheim, Germany, 2018.

ADVANCED MATERIALS

Supporting Information

for *Adv. Mater.*, DOI: 10.1002/adma.201802732

Protein-Corona-by-Design in 2D: A Reliable Platform to Decode Bio–Nano Interactions for the Next-Generation Quality-by-Design Nanomedicines

*Kuo-Ching Mei, Artur Ghazaryan, Er Zhen Teoh, Huw D. Summers, Yueting Li, Belén Ballesteros, Justyna Piasecka, Adam Walters, Robert C. Hider, Volker Mailänder, and Khuloud T. Al-Jamal**

Supporting Information

Protein-corona-by-design in 2D: a reliable platform to decode bio-nano interactions for the next generation quality-by-design nanomedicines

*Kuo-Ching Mei,[1†] Artur Ghazaryan,[2] Er Zhen Teoh,[1] Huw D. Summers,[3] Yueting Li,[1,4] Belén Ballesteros,[5] Justyna Piasecka,[3] Adam Walters,[1] Robert C. Hider,[1] Volker Mailänder,[2,6] Khuloud T. Al-Jamal[1]**

1. Institute of Pharmaceutical Science, Faculty of Life Science & Medicine, King's College London, Franklin-Wilkins Building, 150 Stamford Street, London SE1 9NH, United Kingdom
2. Max Planck Institute for Polymer Research, Ackermannweg 10, 55128 Mainz, Germany
3. Centre for Nanohealth, Swansea University, Singleton Park, Swansea, SA2 8PP
4. Key Laboratory of Pharmaceutics of Guizhou Province, Guizhou Medical University, No. 9, Beijing Road, Yunyan District, Guiyang 550004, China
5. Catalan Institute of Nanoscience and Nanotechnology (ICN2), CSIC and BIST, Campus UAB, Bellaterra, 08193 Barcelona, Spain
6. University Medical Center Mainz, Department of Dermatology, Langenbeckstr. 1, 55131 Mainz, Germany

*** Corresponding Author:** khuloud.al-jamal@kcl.ac.uk

† Present Addresses
Division of NanoMedicine, Center for Environmental Implications of Nanotechnology, California NanoSystems Institute & Department of Medicine, David Geffen School of Medicine, University of California, Los Angeles, 90095 CA, USA

Keywords: *protein corona, graphene, click chemistry, nanomedicine, drug delivery, toxicity*

Supporting Materials and Methods

Materials

Materials for Graphene Synthesis and Functionalisation

Graphite powder (cat: SP-1, batch: 04100, lot: 011705) was obtained from Bay Carbon, Inc. (USA). HCl (cat: 258148), $NaNO_3$, (cat: 221341), $KMnO_4$ (cat: 60459), P_2O_5 (cat: 214701), NaN_3 S2002), 3-(trimethylsilyl)propargyl alcohol (TMS-protected propargyl alcohol, cat: 318558), 4-(Dimethylamino)pyridine (DMAP, cat: 522805) were all obtained from Sigma-Aldrich (UK). 1-Ethyl-3-(3-dimethylaminopropyl)carbodiimide hydrochloride (EDC.HCl cat: ANA29855) was purchased from Bioscience (UK). Silicone oil (cat: A122718, usable ranges 40-200 °C) was from Alfa Aesar (UK). Filter paper (cat: 1001240) was from GE Whatman™ (UK). IsoporeTM membrane, polycarbonate, hydrophilic, 0.22 μm (cat: GTTP04700) were ordered from Merck Millipore (UK). H_2O_2 (cat: 202460010), HNO_3 (cat: 1244660011), and propargyl alcohol (AC131452500) were purchased from ACROS Organics™ (UK). SnackSkin™ dialysis tube, 10K MWCO, 35mm I.D. (cat: 88245) was purchased from ThermoFisher Scientific (UK).

Materials for Protein Corona/SDS-PAGE Analysis and Cell Culture

OXOID phosphate buffered saline tablets (Dulbecco A, cat: BR0014G) was purchased from Fisher Scientific (UK). 1.5mL (cat: E1415-1500), 2 mL (cat: E1420-2000) microcentrifuge tubes were purchased from StarLab (UK). Sodium dodecyl sulfate (SDS, cat: L5750), β-mercaptoethanol (cat: M3148) bromophenol blue (cat: B0126) and Superoxide Dismutase (SOD) determination kit (cat: 19160) were purchased from Sigma (UK). Tris base (cat: TRIS01) was purchased from Formedium (UK). HCl and NaOH (cat: 30620) were purchased from Honeywell Fluka. Glycerol (cat: 444485B) and glycine (cat: 10196X) were purchased from VWR Chemicals (UK). Sucrose (cat: S/8600/60), Triton® X-100 (cat: BP151), 30% w/v

hydrogen peroxide (cat: 10386643) and Dimethyl sulfoxide (DMSO, cat: D/4120/PB08) were purchased from Fisher Scientific (UK). Sterile Newborn Calf Serum (NCS) - heat inactivated (cat: 08-05-850, batch NCS7795) was purchased from First Link Ltd. (UK). Penicillin-Streptomycin (P/S, 10,000 U/mL, 10,000 μg/mL) (cat: 15140122), DMEM-Dulbecco's Modified Eagle Medium-no glucose (cat: 11966025), Advanced RPMI (cat: 12633012), methanol-free 16% w/v formaldehyde (cat: 28908), GlutaMAX™ (cat: 35050061) and trypsin-EDTA (0.25%, phenol red, cat: 25200056) were obtained from ThermoFisher Scientific (UK). J774A.1 (BALB/cN mouse macrophage, ATCC®TIB-67™) were obtained from ATCC®. Calcium fluoride (CaF_2) slide was purchased from Crystran Ltd (UK).

Methods

Graphene Synthesis (Mei's modified Hummers method)

The synthesis of graphene oxide (GO) and clickable graphene derivatives was reported in our previous work. In brief, GO was synthesised using Mei's method (a modified Kovtyukhova-Hummer's method)[1] where the graphite was peroxidised with P_2O_5 and $K_2S_2O_8$ (4:2:2 w/w/w) in H_2SO_4 (1:3 w/v) at 80˚C for 6 h, air-cooled to room temperature then washed with diH_2O by a vacuum flirtation (Millipore® All-Glass 47 mm Vacuum Filter Unit with Isopore™ 0.22 μm hydrophilic polycarbonate filter) until filtrate became pH natural then oven-dried. The pre-oxidised graphite powder was further oxidised using $NaNO_3$ and $KMnO_4$ (1:1:3 w/w/w) in H_2SO_4 (1:5 w/v) through five stages of heating (0-10˚C, 35˚C, 35-60˚C, 60-80˚C, 80˚C) where 50 mL, 50 mL and 80 mL of diH_2O was added to the mixture at the end of stage III, stage IV, and V, respectively. The mixture was kept at 80+˚C for 30 min before adding 35% H_2O_2 (10mL/4g graphite) and mixed for another 30 min to stop the reaction. The black mixture was washed with 1M HCl (1L/4g graphite), 1M NaOH (0.5L/4g graphite), and diH_2O (once) by centrifugation at 3214g for 2 min using 50 mL centrifuge tubes. Supernatants were discarded.

The pellets were re-suspended in diH_2O and sonicated for 1 h using a bath sonicated then centrifuged at 4,000 rpm (Eppendorf 5810R, rotor: A-4-81) for 2 min. Hand-shack the tubes to suspend the loosely packed layer at the top of the pellet, the resulting black dispersion was the GO dispersion. The concentration of the GO dispersion was determined by thermogravimetric analysis.

Graphene Oxide Functionalisation

GO-N_3 was obtained by treating the GO with NaN_3 (1:4 w/w) in water at pH 4.5 for 48 h and dialysed in diH_2O for 72 h (Fisher Snackskin™10kDa 35mm I.D.). GO≡ was obtained by coupling propargyl alcohol with the GO-carboxylate using Steglich esterification by reacting with EDC.HCl and DMAP in water at room temperature for 48 h and dialysed in diH_2O for 72 h. The Click^2 GO (C^2GO, *i.e.* GO with both azide and alkyne/propargyl) was obtained by performing both azide and alkyne (TMS-protected propargyl alcohol) functionalisation sequentially in one pot for 24 + 24 h, centrifuged to remove the reaction solution, re-suspended in diH_2O then dialysed in diH_2O for 72 h to obtain the final product. All compounds were prepared as water dispersion at a concentration at 2.5 mg/mL before used.

Electron Microscopy Imaging of GO & Clickable GO derivatives

GO, and clickable GO derivatives were prepared as stock dispersions at 200 µg/mL in diH_2O and dilute accordingly when needed. GO dispersions were deposited onto copper grids (TEM: carbon film covered, STEM: holey carbon film covered) where excess water was removed by tissue. Sample loaded grids were dried under vacuum before imaging. High resolution transmission electron microscopy (HRTEM) and High angle annular dark field scanning transmission electron microscopy images (HAADF-STEM) were acquired on an FEI Tecnai G2 F20 microscope at 200 kV.

Sample Preparation for Overall Protein Corona Quantification

To prepare 1mg of GO, GO-N_3, GO≡, and C^2GO, 400 μL of the stock solutions (2.5 mg/mL) were transferred into 1.5 mL centrifuge tubes. PBS with 1% penicillin-streptomycin solution (fPBS/PS) was filtered through a 0.22 μm filter and top up the GO dispersions to 1 mL. The graphene PBS dispersions were centrifuged at 10,000 rpm (Eppendorf 5810R, rotor: F45-30-11) for 5 min to precipitate the material, and the supernatants were discarded. The pellet was re-suspended by 1mL fPBS/PS and repeat the centrifuge-washing process for 2 more times to obtain the washed graphene pellets.

Defrosted newborn calf serum (NCS) was transferred into 1.5 mL centrifuge tubes (1.5 mL/each) then centrifuged at 14,000 rpm (Eppendorf 5810R, rotor: F45-30-11), 4˚C, for 60 min. Carefully withdraw 1mL centrifuged serum from the middle layer (avoiding the top and the bottom) from each tube and used the centrifuged cNCS (cNCS) to re-suspend the washed graphene pellets. The cNCS re-suspended graphene dispersions were sonicated for 5 min in a bath sonicator then incubate at 37˚C for 1 h using a shaking water bath.

After the incubation, the 1mL cNCS-Graphene dispersions were transferred into 2 mL centrifuge tubes pre-loaded with 1 mL 0.7M sucrose solution (0.22 μm filtered) then centrifuged at 20,000g for 10 min, rotate the tubes for 180˚ then centrifuge at 14,000 rpm (Eppendorf 5810R, rotor: F45-30-11) for another 10 min. After the centrifugation, cNCS will remain on top of the sucrose layer while the graphene will precipitate to the bottom. cNCS centrifuged on sucrose cushions (scNCS) were isolated (carefully remove 700 μL out of 1 mL and avoid the interface of NCS and the sucrose) and treated as controlled samples, the concentration was measured and treated as 100%. The concentration of scNCS after incubating with GO, GO-N_3, GO≡, and C^2GO were measured, the reduced proteins were assigned as total

protein corona and can be divided into loosely bounded soft corona (SC) and strongly bounded hard corona (HC). The remaining scNCS and the sucrose were removed. The graphene pellets were washed 3 times by re-suspended in 0.5 mL fPBS/PS, centrifuged at 14,000 rpm (Eppendorf 5810R, rotor: F45-30-11) for 10 min and collect the supernatant (0.5 mL x 3 = 1.5 mL/each material). The protein concentrations and quantity of the fPBS/PS washes were measured and regarded as the SC. The HC-graphene pellets (graphene pellets coated with hard corona proteins) were stored at -20˚C before further analysis. To detach the strongly bounded HC for HC quantification, HC-graphene was re-suspended in freshly prepared 2% (w/v) SDS, 62.5m Tris-HCl solution *(*note: this is different from the SDS-PAGE sample/loading buffer, do NOT include bromophenol blue, glycerol and β-mercaptoethanol. See **Table S1** for buffer formulation*).

Protein Quantification using bicinchoninic acid assay (BCA assay) and NanoDrop®

Bicinchoninic acid *assay* (*BCA assay*) and UV based Nanodrop A280 method were compared while searching for serum protein quantification methods suitable for corona studies. BCA assay was carried out in accordance with the manufacturer's protocol (BCA Protein Assay Kit, Pierce Thermo Fisher, Cat#23225). In brief, newborn calf serum (heat inactivated NCS, First Link Ltd., UK) was diluted in phosphate buffered saline (PBS, Invitrogen) in a twofold dilution series from 100% to 0.024% NCS. A volume of 25 µL per analyte was pipetted into each well of a flat bottomed 96 well plate (Corning Costar). PBS was used as a negative control. The working reagent (WR) was prepared by mixing reagent B with reagent A at a 1:50 ratio. To each well, 100 µl of WR was added, the reaction was incubated at 37°C and allowed to proceed for 20 mins before being allowed to cool to room temperature. The optical density (OD) was measured at a wavelength of 562nm using a FLUOstar omega (BMG Labtech). The analysis was performed by subtracting the value obtained for the negative control from the sample

values (n = 4, measured in 2 blocks, i.e. repeated once in a different plate). Nanodrop measurement was straightforward, the analyte (2 µL without further treatment) was deposited on the detector for absorbance read out. One Abs=1 mg/mL was used when measuring protein mixtures as per manufacturer's instruction.

Total protein recovery (by Nanodrop) (shown in **Figure S3C**) was calculated using the following equation:

$$Protein\ Recovery\ (\%) = \frac{HC + SC + post\ incubated\ Sample\ NCS}{Post\ incubated\ Control\ NCS} \times 100\%$$

Hard Corona Preparation for Liquid-chromatography Mass-spectrometry (LC-MS) Analysis

HC-graphene was prepared using the method described in the previous section. To detach the strongly bounded HC, HC-graphene was re-suspended in freshly prepared 2% (w/v) SDS, 62.5m Tris-HCl solution *(*note: this is different from the SDS-PAGE sample/loading buffer, do NOT include bromophenol blue, glycerol and β-mercaptoethanol. See **Table S1** for buffer formulation*). The HC-graphene dispersion was incubated at 95˚C for 5~10 min, then centrifuged at 14000 rpm (Eppendorf 5810R, rotor: F45-30-11), 4 ˚C for 1 h to obtain the clear HC containing supernatant. The protein concentration of the HC solutions was freshly measured by NanoDrop (**Figure 1D**). HC solutions were stored at -80˚C before further analysis.

Hard Corona Profiling

The serum protein in HC solution was digested and analysed with LC-MS using the method described by Schöttler *et al.* (2016) with slight modifications.[2] In brief, 25μg of each HC protein was precipitated and digested by Trypsin (enzyme : protein = 1:50 w/w). The digested peptides were mixed with formic acid (final conc.: 0.1% v/v) and spiked with Hi3 *E.coli* Standard (final conc.: 10 fmol/μL) as internal standards for absolute peptide quantification (injection volume: 2 μL/sample). Quantitative analysis of proteins was carried out using a nanoACQUITY UPLC® system coupled with a Synapt G2-S*i* mass spectrometer (Waters Corporation). A C18 nanoACQUITY Trap Column (5 μm, 180 μm x 20 mm, Waters Corporation) and a C18 analytical reversed-phase column (1.7 μm, 75 μm x 150 mm, Waters Corporation) were used to separate tryptic-digested peptides. A gradient chromatographic separation was performed with ascending acetonitrile (2-40%) in water at a flow rate of 0.3

µL/min for 70 min (all mobile phase contained 0.1% v/v formic acid). A lock-mass reference, Glu-Fibrinopeptide (150 fmol/µL), was co-infused at a flow rate of 0.8 µL/min. Data-independent acquisition (Waters® MS^E) experiments were performed on the Synapt G2-S*i* operated in resolution mode. Electrospray Ionization was performed in positive ion mode using a NanoLockSpray source. Data were acquired over a range of m/z 50-2000 Da with a scan time of 0.5s and a total acquisition time of 80 min. All samples were analysed in two technical replicates and averaged. Data acquisition and processing were carried out using MassLynx™ (v4.1). Progenesis® QI for Proteomics software (v2.0) was used to process data and to identify the proteins. The generated peptide masses were searched against a reviewed human protein sequence databases downloaded from UniProt. Based on the protein sequence obtained from the UniProt, the isoelectric point (pI) and the molecular weight of each HC protein was calculated using the isoelectric point calculator reported by Kozlowski.[3] The sequence-based intrinsic protein solubility was calculated using CamSol Intrinsic software (v2.1).[4] The principal component analysis was performed using Minitab statistical software (v16).

Considerations in protein molar absorption coefficients

From the LC-MS data, it is known that different graphene HC consists of different relative protein abundance %. To verify if such variances in HC RPA will affect the protein molar absorptivity, relative molar absorption coefficient was calculated. As the protein/peptide molar absorption coefficient is related to tryptophan (W), tyrosine (Y), and cysteine (C) residues, the total number of W, Y, and C, was identified for each HC protein, and summed up for each graphene HC based of RPA% using the following equation:

$$nW \text{ or } nY \text{ or } nC \text{ per 100 serum proteins} = \sum_{\#=1}^{30}(Protein_{\#}\ nW \text{ or } nY \text{ or } nC) \times RPA$$

At 280 nm, the wavelength dependent molar absorption coefficient was calculated using the following equation:

$$\varepsilon = (nW \times 5500) + (nY \times 1490) + (nC \times 125)$$

The εGO, εGO-N_3, εGO ≡, εC^2GO were calculated (per 100 proteins basis) and normalised to εGO to evaluate the relative molar absorption coefficients for the 4 HC types. The variability between the HC of the 4 constructs was in the range of 1~2% thus was ignored. The Nanodrop A280 readings were, therefore, used directly for further analysis without additional normalisation (**Figure S4**).

Preparing Graphene Samples for Cell Culture Studies

Graphene water dispersions were transferred into sterilised 1.5 centrifuge tubes. Sterilised PBS containing 5% penicillin-streptomycin (P/S, 500 units/µg/mL), as a poor solvent, was used to top up the volume to 1.5 mL/tube. The PBS buffered graphene dispersion was centrifuged at 14,000 rpm (Eppendorf 5810R, rotor: F45-30-11) for 10 min to precipitate the graphene. The supernatant was removed, and the pellet was re-suspended in sterilised PBS to repeat the washing process for three more times to obtain the washed graphene pellets. The graphene pellets were used directly or coated with protein corona using the methods described before. No fungal contaminations were found by visual inspection when pellets were re-suspended in cell culture media containing 2% penicillin-streptomycin and incubated for 72 h at room temperature followed by 48 h at 37˚C (**Figure S6**).

Overall 72 h Cell Viability Studies via modified LDH assay

J774 cells seeded and were grown in T75 flasks (surface area: 75 cm^2) in DMEM and Adv. RPMI containing 10% NCS and 1% P/S, respectively. When the cells reach 80% confluency,

the cell culture media was removed, and cells were washed with PBS for 3 times before trypsinised at 37˚C for 5 min (5 mL trypsin/T75 flask). The trypsinisation was stopped by adding 5 mL of cell culture media containing 10% NCS. Cells were spin down by centrifugation to remove the trypsin and re-suspended in DMEM/Adv. RPMI containing 10% NCS and 5% P/S before seeding into 96-well cell culture plates. The seeding density for J774 was 6,000 cells/well (0.32 cm^2), respectively, the evening before treating with graphene samples. Graphene samples were freshly washed and prepared in DMEM/Adv. RPMI (10% NCS, 5% P/S) at 1, 50, and 100 μg/mL. The original cell culture media in the 96-well plates were removed and replaced with graphene dispersions and incubated for 72 h (37˚C, 5% CO_2) before cell viability analysis.

The modified lactate dehydrogenase (mLDH) assay was used, *i.e.* instead of measuring the LDH released; the LDH remained within the cell was analysed to minimise the interference from the graphene.[5] In brief, after 72 h incubation, cell culture media was carefully and slowly removed using multi-channel pipettes. J774 cells were semi-adherent cells, which were not washed by PBS to avoid cell lost. The remaining cells were lysed in phenol-red and serum-free DMEM containing 0.9% Triton X-100 at 37˚C for 1h (100 μL/well). The 96-wells plates were there centrifuged at 4,000 rpm (Eppendorf 5810R, rotor: A-4-81) at 4˚C for 1 h to precipitate the graphene within the cell lyse. After the centrifugation, 50 μL/well of the cell lyse was carefully withdrawn from the supernatant (avoiding the graphene precipitate, if any) and transferred into new a 96-well plate. The CytoTox96® LDH assay kit was used for cytotoxicity assay (cat: G1780, Promega, UK). Reconstitute substrate mix was added (50 μL/well), incubated at 37˚C for 15 min before the stop solution was added to terminate the reaction (50 μL/well). The UV absorbance of the reacted cell lyse was read at 490 nm with a microplate

reader (FLUOstar Omega, BMG Labtech) with Omega software (v2.1). The cell viability was calculated using the following equation:

$$Cell\,Viability\,\% = \frac{A_{490}\ of\ Treated\ Cells}{A_{490}\ of\ Untreated\ Cells} \times 100\%$$

72 h Oxidative Stress Analysis via SOD assay for Viable Cells

J774 cells were cultured in DMEM and Adv. RPMI (both with 1% P/S, 1% GlutaMAX™ and 10% NCS), respectively. Cells were maintained in T75 cell culture flask until 80% confluency (37˚C, 5% CO_2) before seeding into 12-well plates. J774 cells were seeded in different 12-well plates (8 x 10^4/well) 24 h before the incubation with graphene samples. Freshly prepared graphene samples (GO, GO-N_3, GO≡, and C^2GO) at 10, 50, and 100 µg/mL were used and incubated with the cells for 72 h (1 mL/well). H_2O_2 was used as a positive control at a concentration of 2.43 mmol/L. After 72 h incubation, ice-chilled PBS was used to gently rinsed the remaining still viable cells (low cell viability at 72 h as showed by the LDH assay). Non-adherent dead cells were aspirated with PBS (1 mL/well). Rinsed viable cells were trypsinised by 300 µL 0.05% trypsin-EDTA at 37˚C for 3 min before adding the serum containing cell culture media to stop the trypsinisation (500 µL/well). Cell suspensions were transferred into 2 mL centrifuge tubes and centrifuged at 4,000 rpm (Beckman Coulter Allegra X-22R Benchtop Centrifuge, rotor: F2402H) for 10 min at 4˚C. The supernatant was discarded. The washing step was repeated twice (1.5 mL PBS/tube). The washed cell pellets were then re-suspended in 200 µL PBS and froze-thawed twice to obtain the cell lyse. The cell lysates were centrifuged at 14,000 rpm (Eppendorf 5810R, rotor: F45-30-11) for 15 min at 4˚C to obtain the supernatant for SOD activity evaluation using SOD determination kit in 96-well plates.

In briefly, three types of blank control samples were prepared: *Blank 1* (20 µL double distilled H_2O + 20µL of enzyme working solution + 200 µL water-soluble tetrazolium/WST working

solution), *Blank 2* (20 µL cell lyse + 20 µL dilution buffer + 200 µL WST working solution), and *Blank 3* (20 µL double distilled H_2O + 20 µL dilution buffer + 200 µL WST working solution). The samples were constituted of cell lyse (20 µL), WST working solution (200 µL) and enzyme working solution (20 µL). The samples and the blank controls were mixed thoroughly within the 96-well plate then incubated at 37 °C for 20 min The UV absorbance of each well was measured at 440 nm using a microplate reader (FLUOstar Omega, BMG Labtech) with Omega software (v2.1). The SOD activity was calculated using the following equation:

$$SOD\ Activity = \frac{(A_{440\ Blank\ 1} - Abs_{440\ Blank\ 3}) - (A_{440\ Sample} - A_{40\ Blank\ 2})}{(A_{440\ Blank\ 1} - A_{450\ Blank\ 3})} \times 100\%$$

The relative SOD activity was calculated by normalising to the untreated control cells.

Cellular Uptake Studies via FlowSight Imaging Cytometer

J774 cells were seeded in T25 cell culture flasks (surface area = 25 cm^2) at a seeding density of 0.7 x 10^6 cells in 10 mL Adv. RPMI (10% NCS, 1% P/S), the day before the experiment. GO, GO-N_3, GO≡, and C^2GO in Adv. RPMI (10% NCS, 5% P/S) was freshly prepared (20 µg/mL, 10 mL) to replace the original cell culture media and incubated with the cells for 24 h. After the incubation, cells were gently washed with PBS once and trypsinised by 3 mL trypsin-ETDA/flask at 37˚C for 5 min then terminated by serum containing media. Cells were centrifuged, and the supernatant was discarded to obtain the black cell pellets (control cell pellet was white). The cell pellet was re-suspended in 4% formaldehyde (w/v) in PBS and fixed at 37˚C for 30 min then washed 3 times with PBS by centrifugation. The washed cell pellets were re-suspended in 50 µL cold PBS in 1.5 mL centrifuge tubes and kept at 4°C prior to flow cytometric analysis.

Cell images were acquired using a FlowSight Imaging cytometer (Merck Millipore), in bright field and dark field configurations. A subset of 3,000 cells taken from the larger cultured cell

population was imaged for each sample and analysed using the manufacturer's software (IDEAS). Linescan gradients were used to select cells within the focal plane, and gating on two-dimensional plots of cell area and aspect ratio were used to select viable cells. The bright field image masking algorithm was used to identify and quantify cell area.

Principal Component Analysis

Principal component analysis (PCA) was performed using Minitab statistical software (v16).

Retrospective Statistical Design of Experiments (DoE)

A retrospective DoE analysis, using historical data, was performed to establish the response surfaces and the predictive model. Raw data that are used to create the Predictive Response Surface for cell viability are summarized in **Table S3**. Data were analysed using Design-Expert 9, v9.0.6.2 (Stat-ease, Inc., USA). Box-cox transformation was performed for the raw data (cell viability %) to improve model fit (power transformed $\lambda = 0 = \log_{10}$ transformation). A suitable predictive model was identified for Factor A (PC-1b), Factor B (PC-2b), and Factor C (graphene dose), using the Sequential Model Sum of Squares (SMSS). The mean square of the model was firstly calculated followed by the addition of a higher-level source of term, i.e., a higher degree of the polynomial in the predictive equation. The aim was to include a higher-level source of terms only if this could explain a significant amount of variation in the responses when compared with the lower-level model. In other words, when one or more predictor variables (source of term) are included in the model, the error sum of squares (SSE) should be reduced, or the regression sum of square (SSR) should be increased. As shown in **Table S4**, the linear Model F value of 190.29 implied the model was significant that explained a significant amount of variability in the responses when compared to the overall sample mean (p-value < 0.0001). The lack of fit for linear model was not significant, indicating a good fit (p-value = 0.0607, **Table S5**) The "Predicted R^2 " of 0.9153 was in reasonable agreement with

the "Adjusted R^2 " of 0.9236, *i.e.,* the difference is less than 0.2. (**Table S6**) The ANOVA table for the 3-factor linear model is shown in **Table S7**, where all three factors are significant (p-value < 0.001). "Adeq. Precision" of 42.436 indicates an adequate signal, *i.e.,* a good signal-to-noise ratio. This model could be used to navigate the design space. The coefficients of the model terms are shown in **Table S8** with a small Variance Inflation Factor (VIF) of 1 for all factors.

Supporting Results

Protein Quantification using bicinchoninic acid assay (BCA assay) and NanoDrop®

As shown in **Figure S3A**, while BCA assay saturated before the theoretical quantification limit (2 mg/mL = 2.17% serum), the actual reliable and reproducible quantification range sits at <1% serum = 0.89 mg/mL. Noting that BCA assay is a non-terminating assay, i.e. the inter- and intra- day/lab/person reproducibility will be low, the variance was also high with ~15% coefficient of variances, at concentrations approaching 2 mg/mL, even when the timing is perfectly controlled. Nanodrop reliably and linearly measured concentrations as low as 0.024 % serum (0.03 ± 0.01 mg/mL) and up to 100% serum (68.29 ± 0.30 mg/mL) (**Figure S3B**). It was crucial that the method of choice covers a linear range up to the 100% protein recovery value, ideally without the need for additional dilutions steps for better recovery value accuracy. All graphene samples showed a 98-99% protein recovery by the nanodrop method, indicating that the isolation of HC and SC was successful and validating the nanodrop method. Nanodrop was therefore selected as the preferred protein quantification method as has been previously published.[6]

Supporting Figures and Tables

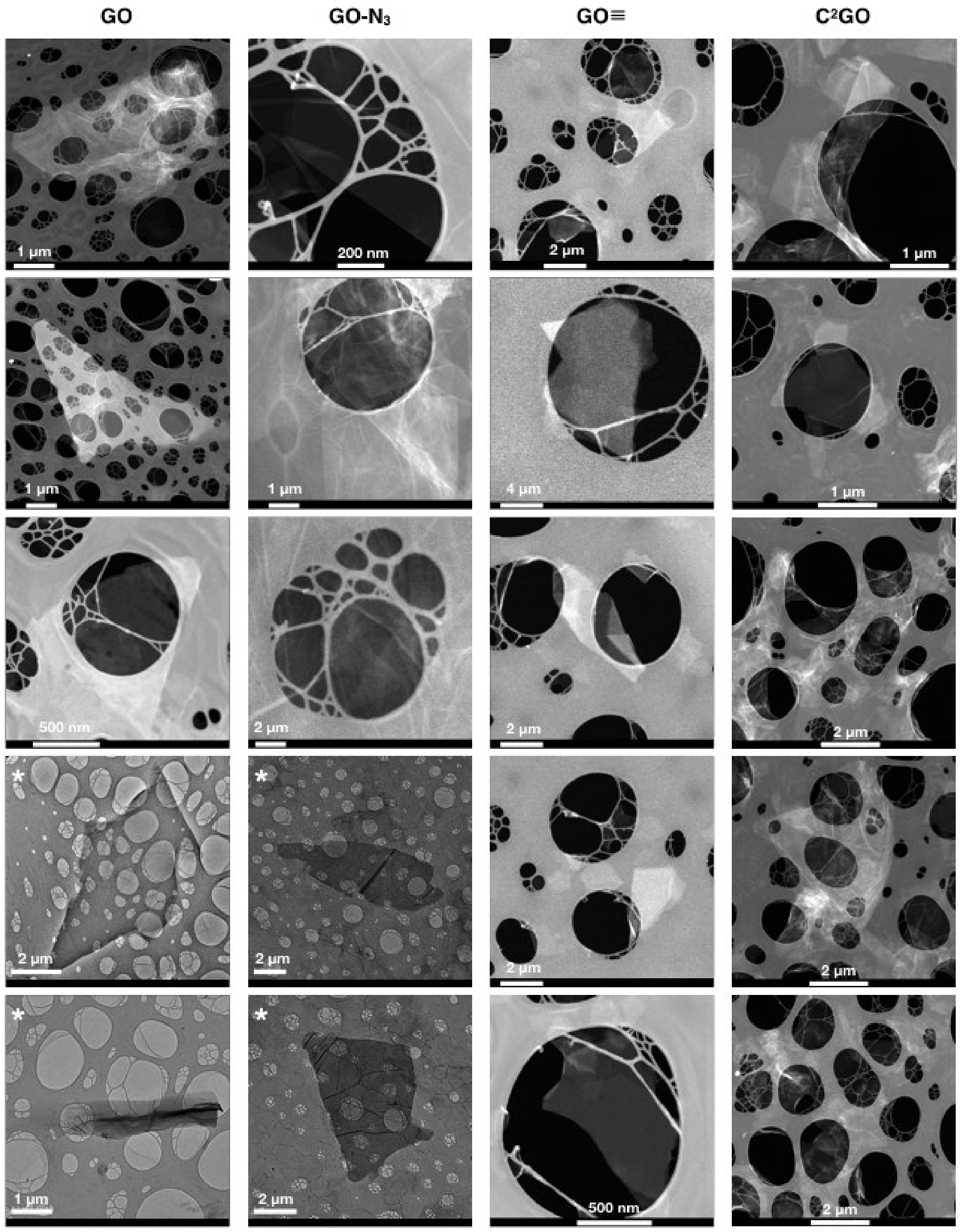

Figure S1. Electron microscopy images. STEM and TEM (images labelled with *) are shown for GO, GO-N_3, GO≡, and C^2GO. Film-like structures were confirmed for all GO derivatives regardless of the surface functionalisation.

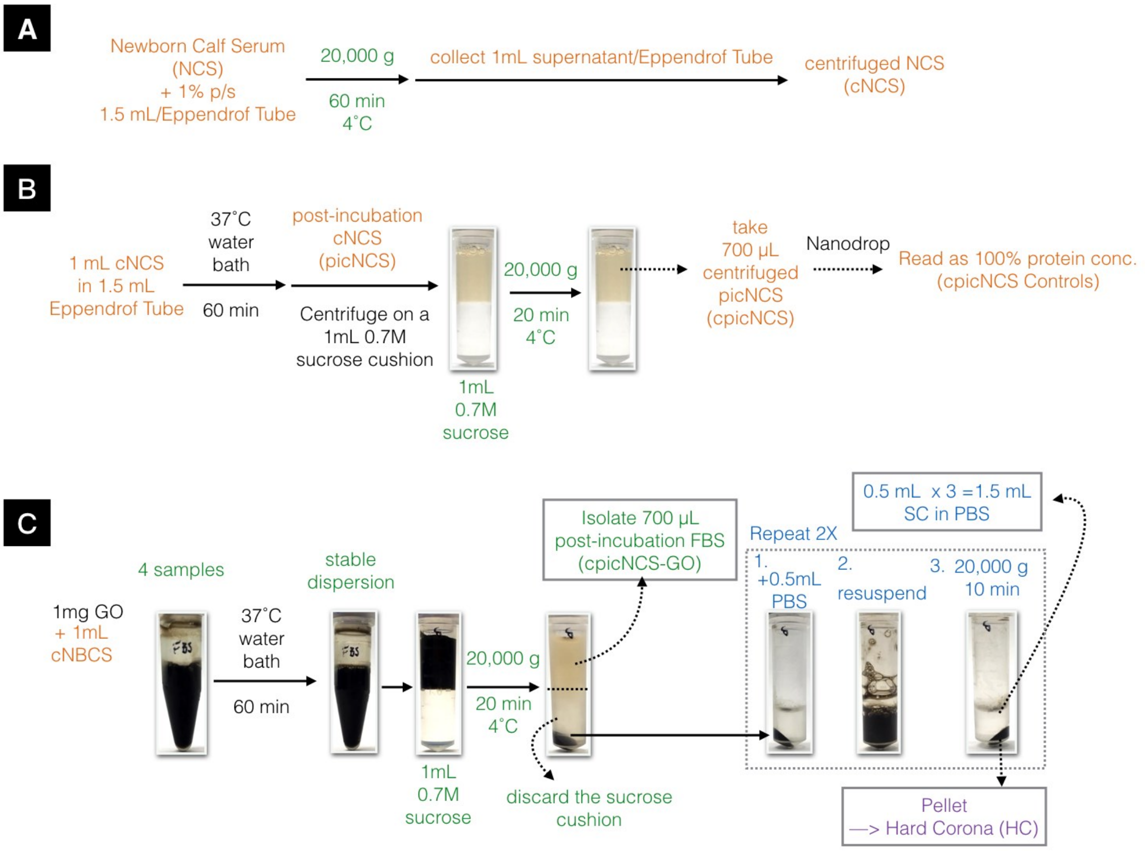

Figure S2. Schematic work-flow for protein corona studies. A) Newborn calf serum was first centrifuged at 20,000 g for 60 min to collect the supernatant (cNCS). **B)** cNCS was incubated at 37˚C for 60 min then centrifuged on top of a sucrose cushion at 20,000 g for 20 min at 4˚C. The protein concentration of the final centrifuged, post-incubation cNCS (cpicNCS) was treated as control samples. **C).** One mg of the graphene was incubated with 1mL of cNCS at 37˚C for 60 min in a shaking water bath. The black dispersion was loaded on top of a sucrose cushion and centrifuged at 20,000 g for 20 min at 4˚C to precipitate the hard corona coated graphene. The cpicNCS protein concentration of GO, GO-N_3, GO≡, and C^2GO were measured to identify the total protein lost due to the corona formation. The sucrose gradient was discarded, and the graphene pellet was washed 3 times with PBS to isolated the SC, leaving the HC coated graphene pellets to be stored at – 20˚C before further analysis.

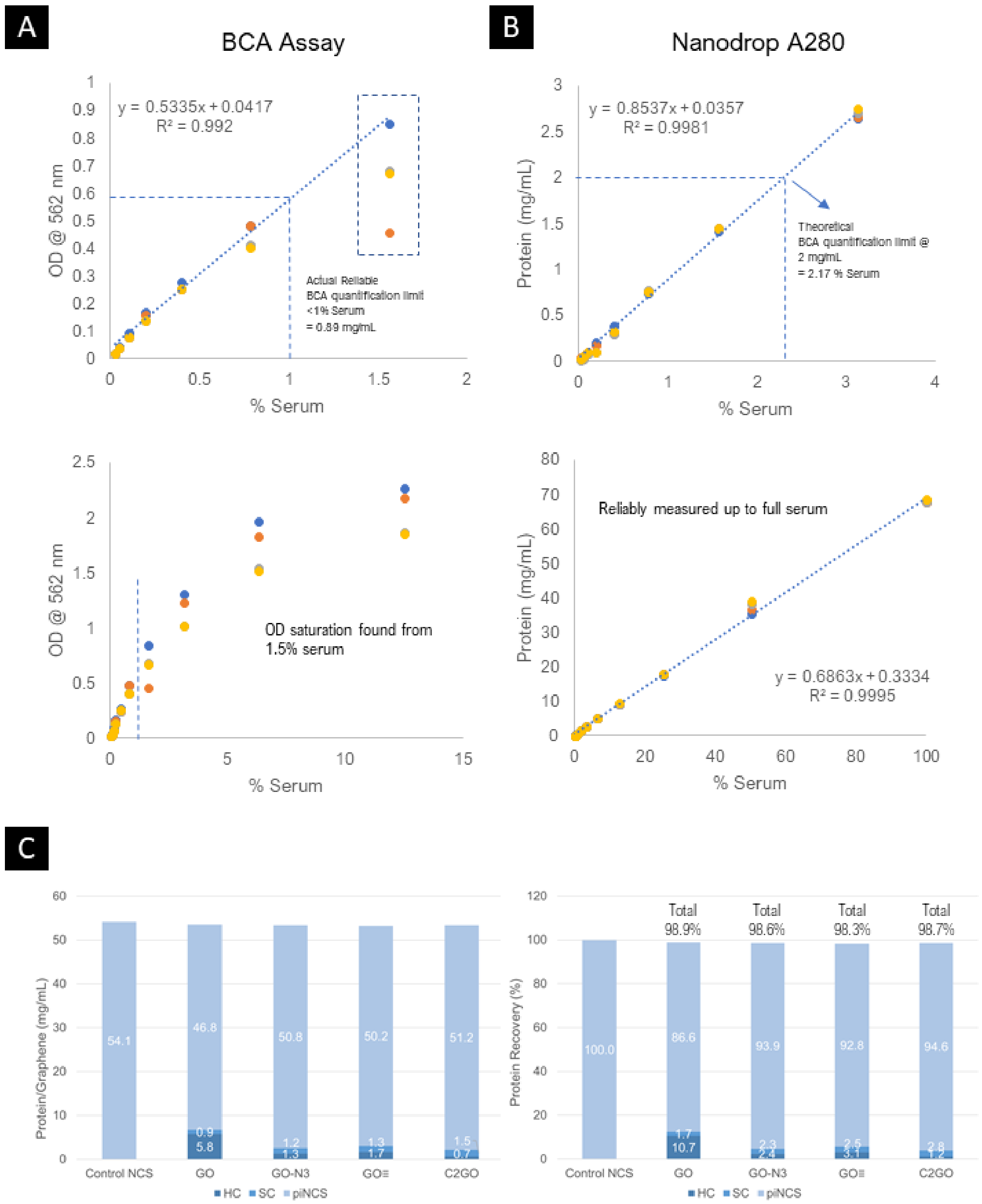

Figure S3. Serum protein quantification using BCA assay **(A)** and Nanodrop **(B). (C)** A 98+% total serum protein recovery was found for all samples.

Protein #		No. of Amino Acid per Protein			Relative Protein Abundance (RPA) % (numbers of proteins / 100 HC serum proteins)			
		Trypton (W)	Tysosin (Y)	Cysteine (C)	GO	GO-N_3	GO≡	C^2GO
1	Serum Albumin	14	21	24	12.7	15.1	15.9	15.8
2	Serotransferrin	8	25	40	11.3	8.7	7.8	8.1
3	Inter-alpha-trypsin Inhibitor Heavy Chain H2	9	27	8	4.9	5.0	5.9	7.0
4	Beta-actin-like Protein 2	4	16	6	11.1	9.4	9.3	8.2
5	Aplha-2-HS-Glycoprotein	3	7	14	10.2	8.5	9.6	8.7
6	Vitamin D Binding Protein	1	16	28	2.7	2.4	3.1	3.3
7	Apolipoprotein A-I	5	7	0	3.1	3.3	3.4	2.9
8	Lactotransferrin	10	21	33	0.8	0.9	0.9	0.9
9	Hemoglobin Subunit Alpha	1	3	1	12.1	13.1	12.5	13.7
10	POTE Ankyrin Domain Family Member F	10	28	29	3.3	2.8	2.5	3.0
11	Complement C3	17	57	27	2.4	2.0	2.3	2.1
12	Alpha-2 Macroglobulin	11	55	25	3.9	4.6	4.8	4.1
13	Prothrombin	14	21	26	0.6	0.8	0.6	0.5
14	Hemoglobin Subunit Epsilon	3	2	1	3.5	3.4	2.9	2.6
15	Beta-2 Glycoprotein 1	5	14	23	2.9	3.9	3.4	5.2
16	Inter-alpha-trypsin Inhibitor Heavy Chain H4	10	21	4	3.0	2.8	2.9	2.6
17	Antithrombin-III	5	12	8	1.0	1.1	1.0	0.9
18	Pregnancy Zone Protein	11	48	26	0.8	0.8	0.7	0.6
19	Complement C9	4	21	26	1.2	1.4	1.4	1.1
20	Tetranectin	5	5	7	0.4	0.5	0.5	0.6
21	Hemoglobin Subunit Delta	2	3	2	1.3	1.5	1.4	1.1
22	Ig Heavy Constant Gamma-1	5	12	9	0.5	0.6	0.6	0.6
23	Apolipoprotein C-III	3	2	0	0.8	1.2	1.0	0.9
24	Ig Lambda Constant 3	2	4	3	0.3	0.3	0.3	0.3
25	Ig Kappa Constant	1	4	3	0.3	0.4	0.4	0.4
26	Thrombospondin-1	22	31	70	0.9	1.2	1.1	1.3
27	Gelsolin	15	22	7	0.5	0.7	0.6	0.6
28	Vitronectin	10	23	14	0.7	0.8	0.6	0.7
29	Actin, Cytoplasmic 2	4	15	6	0.5	0.5	0.6	0.5
30	Hemoglobin Subunit Beta	2	3	2	0.6	0.7	0.7	0.5

Amino Acid Numbers in Hard Corona per 100 proteins = W/Y/C per protein x RPA

Protein #	GO			GO-N_3			GO≡			C^2GO		
	W	Y	C	W	Y	C	W	Y	C	W	Y	C
1	178.1	267.1	305.3	211.8	317.7	363.1	222.5	333.7	381.4	221.1	331.6	379.0
2	90.5	282.8	452.4	69.8	218.0	348.8	62.0	193.8	310.0	65.0	203.3	325.2
3	44.3	132.8	39.4	44.8	134.5	39.8	52.8	158.5	47.0	62.6	187.7	55.6
4	44.2	177.0	66.4	37.5	149.9	56.2	37.4	149.4	56.0	32.8	131.2	49.2
5	30.5	71.1	142.1	25.4	59.4	118.7	28.7	66.9	133.7	26.1	60.9	121.8
6	2.7	42.6	74.5	2.4	38.1	66.6	3.1	49.0	85.7	3.3	52.2	91.3
7	15.7	22.0	0.0	16.5	23.1	0.0	16.8	23.5	0.0	14.3	20.0	0.0
8	8.1	17.0	26.7	9.1	19.1	30.0	8.6	18.1	28.4	8.5	17.9	28.1
9	12.1	36.4	12.1	13.1	39.2	13.1	12.5	37.4	12.5	13.7	41.0	13.7
10	33.4	93.5	96.9	27.5	77.0	79.8	25.1	70.3	72.8	29.7	83.2	86.1
11	40.8	136.8	64.8	34.3	115.1	54.5	39.4	132.2	62.6	35.4	118.6	56.2
12	42.7	213.4	97.0	50.6	253.0	115.0	52.7	263.5	119.8	44.9	224.4	102.0
13	9.0	13.4	16.6	10.5	15.8	19.6	8.7	13.0	16.1	7.4	11.2	13.8
14	10.6	7.1	3.5	10.2	6.8	3.4	8.8	5.9	2.9	7.7	5.1	2.6
15	14.5	40.5	66.5	19.4	54.2	89.0	17.2	48.2	79.1	26.2	73.2	120.3
16	29.5	62.0	11.8	27.8	58.4	11.1	28.7	60.3	11.5	26.4	55.4	10.6
17	4.9	11.6	7.8	5.5	13.1	8.7	4.8	11.5	7.7	4.7	11.3	7.5
18	9.2	40.0	21.7	8.3	36.0	19.5	7.8	33.9	18.4	6.9	30.1	16.3
19	4.7	24.8	30.7	5.6	29.2	36.1	5.4	28.6	35.4	4.3	22.7	28.1
20	2.2	2.2	3.1	2.7	2.7	3.8	2.6	2.6	3.7	2.9	2.9	4.0
21	2.5	3.8	2.5	3.0	4.5	3.0	2.7	4.1	2.7	2.2	3.2	2.2
22	2.6	6.3	4.7	3.1	7.4	5.5	2.9	7.1	5.3	2.9	6.9	5.2
23	2.5	1.7	0.0	3.5	2.4	0.0	2.9	1.9	0.0	2.6	1.7	0.0
24	0.5	1.1	0.8	0.6	1.2	0.9	0.6	1.1	0.8	0.6	1.2	0.9
25	0.3	1.1	0.8	0.4	1.7	1.3	0.4	1.6	1.2	0.4	1.6	1.2
26	19.4	27.3	61.6	26.6	37.5	84.7	24.2	34.1	77.0	27.7	39.1	88.2
27	7.6	11.1	3.5	10.1	14.8	4.7	8.7	12.7	4.1	8.5	12.5	4.0
28	6.6	15.2	9.3	8.3	19.2	11.7	5.7	13.2	8.0	6.7	15.4	9.4
29	2.1	7.8	3.1	2.1	7.7	3.1	2.4	8.9	3.6	2.1	7.7	3.1
30	1.2	1.9	1.2	1.5	2.2	1.5	1.3	2.0	1.3	1.1	1.6	1.1

	GO			GO-N_3			GO≡			C^2GO		
SUM or amino acid numbers per 100 serum proteins	672.8	1771.1	1626.7	691.8	1758.7	1593.3	697.2	1786.7	1588.5	698.4	1774.6	1626.4
ε (molar absorption coefficient @ 280 nm) = (nW × 5500) + (nY × 1490) + (nC × 125)=	6.54E+06			6.62E+06			6.70E+06			6.69E+06		
Relative ε % Normalised to GO	100%			101%			102%			102%		

Figure S4. The relative molar absorption coefficient for different HC samples

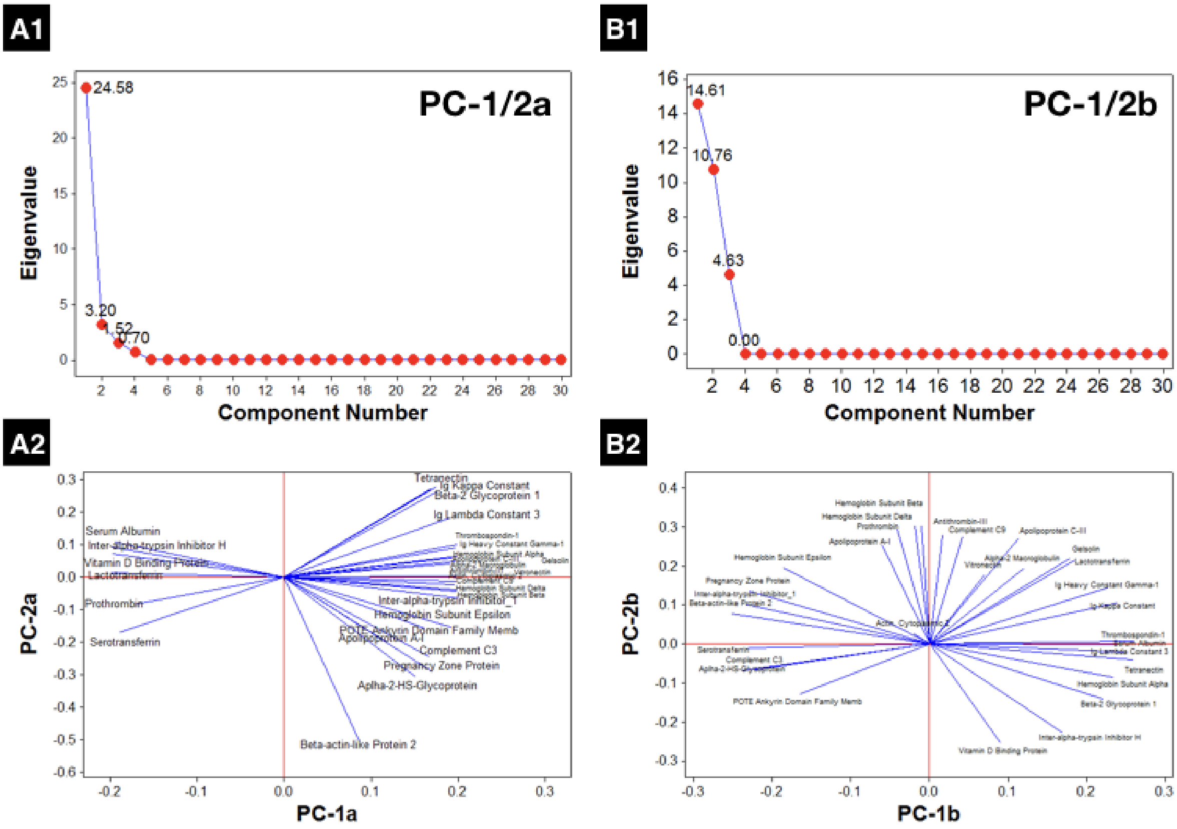

Figure S5. The screening (Eigenvalue) and loading (Eigenvector) plots of the principal component analysis showed in **Figure 2.**

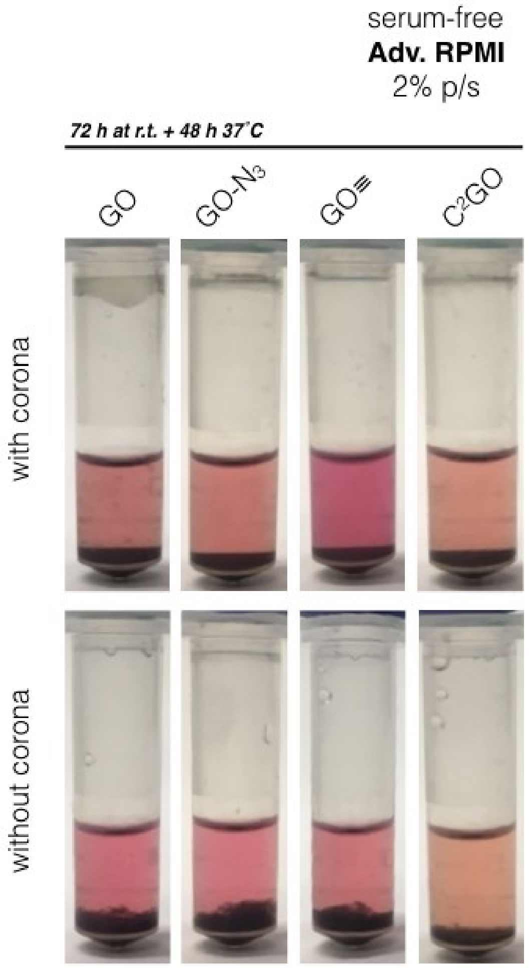

Figure S6. Graphene sample preparation for cell culture studies. Graphene was synthesised in strong acids, the exfoliation process, however, was performed in a non-sterile environment. GO dispersion blocked 0.22 µm filter instantly, *i.e.* cannot be sterilised by filtration. The heat and pressure generated by the autoclaving process may also change the surface chemistry of the graphene, therefore, was not selected for graphene sterilisation. To prepared clean graphene for cell-culture studies, graphene was first mixed with sterilised PBS containing 2% penicillin-streptomycin in the sterilised bio-safety hood then centrifuged at 15,000 g to precipitate. The graphene pellet was re-suspended, repeatedly washed by centrifugation for 3 more times and finally re-suspended in cell culture media containing 2% penicillin-streptomycin (4 washes in total). Figured showed the graphene with or without HC in RMPI (cell culture media for J774 cells), at a graphene concentration of 100 µg/mL. All cell culture media remain clear without being turbid (a sign of contamination) after 72 h at room temperature followed by 48 h at 37˚C. The washing process was then adapted for sample preparation for cell culture studies

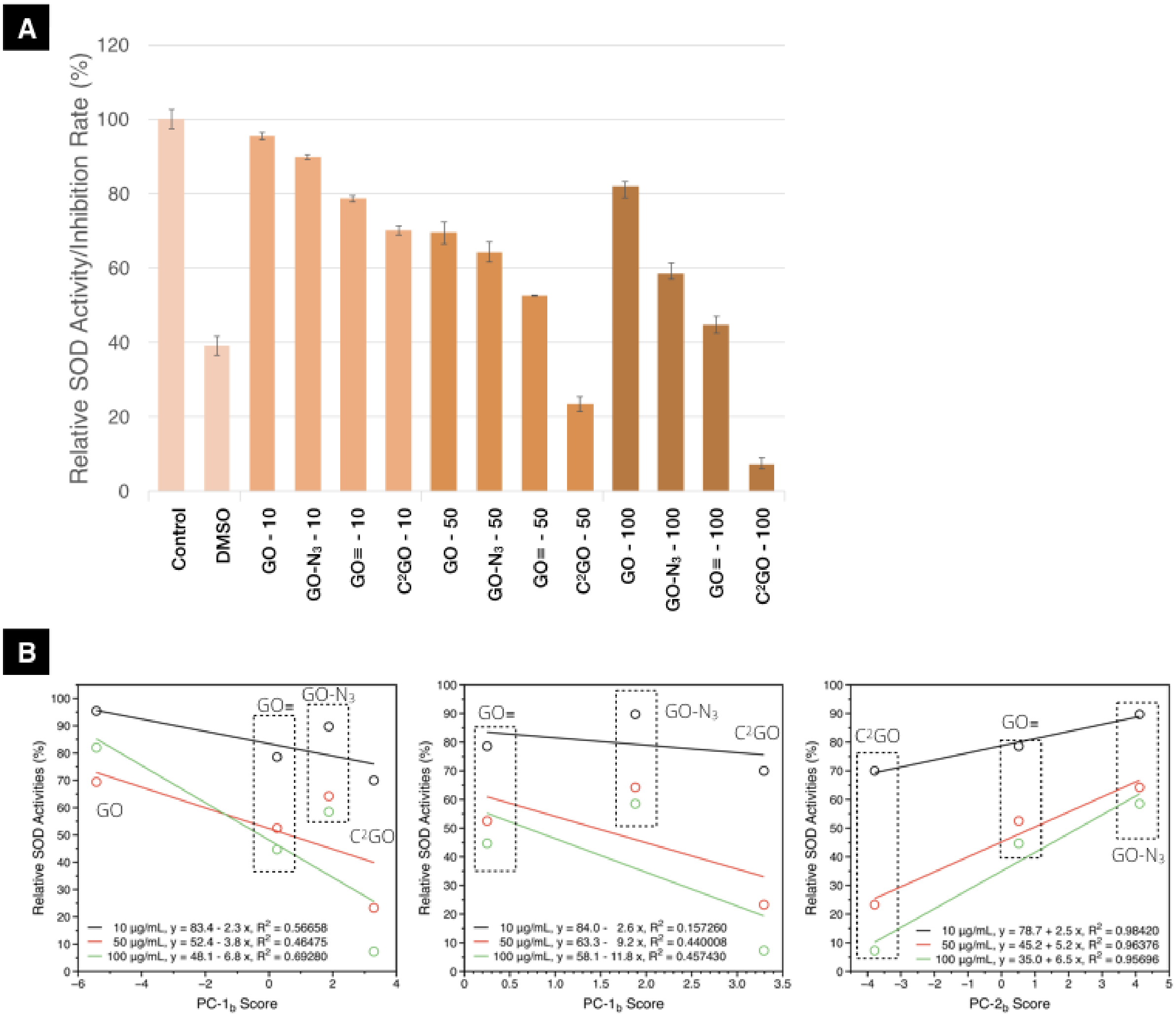

Figure S7. Relative SOD activity studies for viable J774 cells after 72h incubation.

	M.W.	pI	I.O.S.S.	PC-1a	PC-2a	PC-1b	PC-2b	Serum	GO	GO-N3	GO≡	C2GO
Apolipoprotein C-III	10.9	5.1	0.9	0.19	0.06	0.11	0.27	0.3	0.8	1.2	1.0	0.9
Ig Lambda Constant 3	11.3	6.5	1.0	0.18	0.26	0.11	0.27	0.2	0.3	0.3	0.3	0.3
Ig Kappa Constant	11.8	5.8	0.7	0.17	0.27	0.24	0.11	0.2	0.3	0.4	0.4	0.4
Hemoglobin Subunit Alpha	15.3	8.1	0.5	0.20	0.06	0.23	-0.09	1.9	12.1	13.1	12.5	13.7
Hemoglobin Subunit Beta	16.0	6.5	0.2	0.20	-0.04	-0.01	0.30	0.1	0.6	0.7	0.7	0.5
Hemoglobin Subunit Delta	16.1	7.4	0.4	0.20	-0.04	-0.02	0.30	0.3	1.3	1.5	1.4	1.1
Hemoglobin Subunit Epsilon	16.2	8.0	0.1	0.19	-0.15	-0.19	0.19	0.8	3.5	3.4	2.9	2.6
Tetranectin	22.5	5.4	0.2	0.17	0.28	0.26	-0.04	0.3	0.4	0.5	0.5	0.6
Apolipoprotein A-I	30.8	5.4	1.7	0.11	-0.19	-0.06	0.25	2.9	3.1	3.3	3.4	2.9
Ig Heavy Constant Gamma-1	36.1	7.4	-0.1	0.20	0.10	0.23	0.14	0.3	0.5	0.6	0.6	0.6
Beta-2 Glycoprotein 1	38.3	7.1	0.1	0.17	0.27	0.22	-0.14	0.7	2.9	3.9	3.4	5.2
Aplha-2-HS-Glycoprotein	39.3	5.2	0.5	0.15	-0.31	-0.24	-0.07	7.4	10.2	8.5	9.6	8.7
Actin, Cytoplasmic 2	41.8	5.2	0.4	0.20	-0.01	-0.02	0.03	0.1	0.5	0.5	0.6	0.5
Beta-actin-like Protein 2	42.0	5.2	0.3	0.09	-0.50	-0.25	0.08	8.4	11.1	9.4	9.3	8.2
Antithrombin-III	52.6	6.1	0.2	0.20	0.00	0.04	0.28	0.6	1.0	1.1	1.0	0.9
Vitamin D Binding Protein	53.0	5.3	0.8	-0.20	0.07	0.09	-0.25	5.4	2.7	2.4	3.1	3.3
Vitronectin	54.3	5.4	1.4	0.19	0.04	0.07	0.18	0.2	0.7	0.8	0.6	0.7
Complement C9	63.2	5.3	0.8	0.20	-0.02	0.02	0.28	0.5	1.2	1.4	1.4	1.1
Serum Albumin	69.4	5.7	0.5	-0.19	0.11	0.24	0.00	27.5	12.7	15.1	15.9	15.8
Prothrombin	70.0	5.4	0.7	-0.17	-0.08	-0.04	0.29	1.0	0.6	0.8	0.6	0.5
Serotransferrin	77.1	6.3	0.3	-0.19	-0.17	-0.23	-0.01	17.0	11.3	8.7	7.8	8.1
Lactotransferrin	78.2	7.2	0.3	-0.20	0.01	0.18	0.22	2.7	0.8	0.9	0.9	0.9
Gelsolin	85.7	5.7	0.7	0.20	0.09	0.18	0.22	0.2	0.5	0.7	0.6	0.6
Inter-alpha-trypsin Inhibitor Heavy Chain H4	103.4	6.2	-0.7	0.20	-0.06	-0.23	0.10	0.7	3.0	2.8	2.9	2.6
Inter-alpha-trypsin Inhibitor Heavy Chain H2	106.5	6.1	-1.1	-0.20	0.09	0.17	-0.23	13.9	4.9	5.0	5.9	7.0
POTE Ankyrin Domain Family Member F	121.4	5.6	1.7	0.16	-0.16	-0.16	-0.13	1.9	3.3	2.8	2.5	3.0
Thrombospondin-1	129.4	4.6	1.1	0.19	0.18	0.26	0.01	0.2	0.9	1.2	1.1	1.3
Alpha-2 Macroglobulin	163.3	5.8	-2.9	0.20	0.05	0.12	0.19	1.5	3.9	4.6	4.8	4.1
Pregnancy Zone Protein	163.9	5.7	-3.4	0.17	-0.29	-0.24	0.14	0.5	0.8	0.8	0.7	0.6
Complement C3	187.2	5.8	-1.8	0.17	-0.24	-0.22	-0.07	1.6	2.4	2.0	2.3	2.1

Figure S8. Protein MW sorted against pI, I.O.S.S., PCs, and HC RPA.

	M.W.	pI	I.O.S.S.	PC-1a	PC-2a	PC-1b	PC-2b	Serum	GO	GO-N3	GO≡	C2GO
Thrombospondin-1	129.4	4.6	1.1	0.19	0.18	0.26	0.01	0.2	0.9	1.2	1.1	1.3
Apolipoprotein C-III	10.9	5.1	0.9	0.19	0.06	0.11	0.27	0.3	0.8	1.2	1.0	0.9
Actin, Cytoplasmic 2	41.8	5.2	0.4	0.20	-0.01	-0.02	0.03	0.1	0.5	0.5	0.6	0.5
Aplha-2-HS-Glycoprotein	39.3	5.2	0.5	0.15	-0.31	-0.24	-0.07	7.4	10.2	8.5	9.6	8.7
Beta-actin-like Protein 2	42.0	5.2	0.3	0.09	-0.50	-0.25	0.08	8.4	11.1	9.4	9.3	8.2
Vitamin D Binding Protein	53.0	5.3	0.8	-0.20	0.07	0.09	-0.25	5.4	2.7	2.4	3.1	3.3
Complement C9	63.2	5.3	0.8	0.20	-0.02	0.02	0.28	0.5	1.2	1.4	1.4	1.1
Vitronectin	54.3	5.4	1.4	0.19	0.04	0.07	0.18	0.2	0.7	0.8	0.6	0.7
Tetranectin	22.5	5.4	0.2	0.17	0.28	0.26	-0.04	0.3	0.4	0.5	0.5	0.6
Apolipoprotein A-I	30.8	5.4	1.7	0.11	-0.19	-0.06	0.25	2.9	3.1	3.3	3.4	2.9
Prothrombin	70.0	5.4	0.7	-0.17	-0.08	-0.04	0.29	1.0	0.6	0.8	0.6	0.5
POTE Ankyrin Domain Family Member F	121.4	5.6	1.7	0.16	-0.16	-0.16	-0.13	1.9	3.3	2.8	2.5	3.0
Serum Albumin	69.4	5.7	0.5	-0.19	0.11	0.24	0.00	27.5	12.7	15.1	15.9	15.8
Gelsolin	85.7	5.7	0.7	0.20	0.09	0.18	0.22	0.2	0.5	0.7	0.6	0.6
Pregnancy Zone Protein	163.9	5.7	-3.4	0.17	-0.29	-0.24	0.14	0.5	0.8	0.8	0.7	0.6
Alpha-2 Macroglobulin	163.3	5.8	-2.9	0.20	0.05	0.12	0.19	1.5	3.9	4.6	4.8	4.1
Complement C3	187.2	5.8	-1.8	0.17	-0.24	-0.22	-0.07	1.6	2.4	2.0	2.3	2.1
Ig Kappa Constant	11.8	5.8	0.7	0.17	0.27	0.24	0.11	0.2	0.3	0.4	0.4	0.4
Antithrombin-III	52.6	6.1	0.2	0.20	0.00	0.04	0.28	0.6	1.0	1.1	1.0	0.9
Inter-alpha-trypsin Inhibitor Heavy Chain H2	106.5	6.1	-1.1	-0.20	0.09	0.17	-0.23	13.9	4.9	5.0	5.9	7.0
Inter-alpha-trypsin Inhibitor Heavy Chain H4	103.4	6.2	-0.7	0.20	-0.06	-0.23	0.10	0.7	3.0	2.8	2.9	2.6
Serotransferrin	77.1	6.3	0.3	-0.19	-0.17	-0.23	-0.01	17.0	11.3	8.7	7.8	8.1
Hemoglobin Subunit Beta	16.0	6.5	0.2	0.20	-0.04	-0.01	0.30	0.1	0.6	0.7	0.7	0.5
Ig Lambda Constant 3	11.3	6.5	1.0	0.18	0.26	0.11	0.27	0.2	0.3	0.3	0.3	0.3
Beta-2 Glycoprotein 1	38.3	7.1	0.1	0.17	0.27	0.22	-0.14	0.7	2.9	3.9	3.4	5.2
Lactotransferrin	78.2	7.2	0.3	-0.20	0.01	0.18	0.22	2.7	0.8	0.9	0.9	0.9
Ig Heavy Constant Gamma-1	36.1	7.4	-0.1	0.20	0.10	0.23	0.14	0.3	0.5	0.6	0.6	0.6
Hemoglobin Subunit Delta	16.1	7.4	0.4	0.20	-0.04	-0.02	0.30	0.3	1.3	1.5	1.4	1.1
Hemoglobin Subunit Epsilon	16.2	8.0	0.1	0.19	-0.15	-0.19	0.19	0.8	3.5	3.4	2.9	2.6
Hemoglobin Subunit Alpha	15.3	8.1	0.5	0.20	0.06	0.23	-0.09	1.9	12.1	13.1	12.5	13.7

Figure S9. Protein pI sorted against MW, I.O.S.S., PCs, and HC RPA.

	M.W.	pI	I.O.S.S.	PC-1a	PC-2a	PC-1b	PC-2b	Serum	GO	GO-N3	GO≡	C2GO
Pregnancy Zone Protein	163.9	5.7	-3.4	0.17	-0.29	-0.24	0.14	0.5	0.8	0.8	0.7	0.6
Alpha-2 Macroglobulin	163.3	5.8	-2.9	0.20	0.05	0.12	0.19	1.5	3.9	4.6	4.8	4.1
Complement C3	187.2	5.8	-1.8	0.17	-0.24	-0.22	-0.07	1.6	2.4	2.0	2.3	2.1
Inter-alpha-trypsin Inhibitor Heavy Chain H2	106.5	6.1	-1.1	-0.20	0.09	0.17	-0.23	13.9	4.9	5.0	5.9	7.0
Inter-alpha-trypsin Inhibitor Heavy Chain H4	103.4	6.2	-0.7	0.20	-0.06	-0.23	0.10	0.7	3.0	2.8	2.9	2.6
Ig Heavy Constant Gamma-1	36.1	7.4	-0.1	0.20	0.10	0.23	0.14	0.3	0.5	0.6	0.6	0.6
Hemoglobin Subunit Epsilon	16.2	8.0	0.1	0.19	-0.15	-0.19	0.19	0.8	3.5	3.4	2.9	2.6
Beta-2 Glycoprotein 1	38.3	7.1	0.1	0.17	0.27	0.22	-0.14	0.7	2.9	3.9	3.4	5.2
Antithrombin-III	52.6	6.1	0.2	0.20	0.00	0.04	0.28	0.6	1.0	1.1	1.0	0.9
Tetranectin	22.5	5.4	0.2	0.17	0.28	0.26	-0.04	0.3	0.4	0.5	0.5	0.6
Hemoglobin Subunit Beta	16.0	6.5	0.2	0.20	-0.04	-0.01	0.30	0.1	0.6	0.7	0.7	0.5
Serotransferrin	77.1	6.3	0.3	-0.19	-0.17	-0.23	-0.01	17.0	11.3	8.7	7.8	8.1
Beta-actin-like Protein 2	42.0	5.2	0.3	0.09	-0.50	-0.25	0.08	8.4	11.1	9.4	9.3	8.2
Lactotransferrin	78.2	7.2	0.3	-0.20	0.01	0.18	0.22	2.7	0.8	0.9	0.9	0.9
Hemoglobin Subunit Delta	16.1	7.4	0.4	0.20	-0.04	-0.02	0.30	0.3	1.3	1.5	1.4	1.1
Actin, Cytoplasmic 2	41.8	5.2	0.4	0.20	-0.01	-0.02	0.03	0.1	0.5	0.5	0.6	0.5
Aplha-2-HS-Glycoprotein	39.3	5.2	0.5	0.15	-0.31	-0.24	-0.07	7.4	10.2	8.5	9.6	8.7
Hemoglobin Subunit Alpha	15.3	8.1	0.5	0.20	0.06	0.23	-0.09	1.9	12.1	13.1	12.5	13.7
Serum Albumin	69.4	5.7	0.5	-0.19	0.11	0.24	0.00	27.5	12.7	15.1	15.9	15.8
Ig Kappa Constant	11.8	5.8	0.7	0.17	0.27	0.24	0.11	0.2	0.3	0.4	0.4	0.4
Gelsolin	85.7	5.7	0.7	0.20	0.09	0.18	0.22	0.2	0.5	0.7	0.6	0.6
Prothrombin	70.0	5.4	0.7	-0.17	-0.08	-0.04	0.29	1.0	0.6	0.8	0.6	0.5
Vitamin D Binding Protein	53.0	5.3	0.8	-0.20	0.07	0.09	-0.25	5.4	2.7	2.4	3.1	3.3
Complement C9	63.2	5.3	0.8	0.20	-0.02	0.02	0.28	0.5	1.2	1.4	1.4	1.1
Apolipoprotein C-III	10.9	5.1	0.9	0.19	0.06	0.11	0.27	0.3	0.8	1.2	1.0	0.9
Ig Lambda Constant 3	11.3	6.5	1.0	0.18	0.26	0.11	0.27	0.2	0.3	0.3	0.3	0.3
Thrombospondin-1	129.4	4.6	1.1	0.19	0.18	0.26	0.01	0.2	0.9	1.2	1.1	1.3
Vitronectin	54.3	5.4	1.4	0.19	0.04	0.07	0.18	0.2	0.7	0.8	0.6	0.7
POTE Ankyrin Domain Family Member F	121.4	5.6	1.7	0.16	-0.16	-0.16	-0.13	1.9	3.3	2.8	2.5	3.0
Apolipoprotein A-I	30.8	5.4	1.7	0.11	-0.19	-0.06	0.25	2.9	3.1	3.3	3.4	2.9

Figure S10. Protein I.O.S.S. sorted against MW, pI, PCs, and HC RPA.

	M.W.	pI	I.O.S.S.	PC-1a	PC-2a	PC-1b	PC-2b	Serum	GO	GO-N3	GO≡	C2GO
Inter-alpha-trypsin Inhibitor Heavy Chain H2	106.5	6.1	-1.1	-0.20	0.09	0.17	-0.23	13.9	4.9	5.0	5.9	7.0
Lactotransferrin	78.2	7.2	0.3	-0.20	0.01	0.18	0.22	2.7	0.8	0.9	0.9	0.9
Vitamin D Binding Protein	53.0	5.3	0.8	-0.20	0.07	0.09	-0.25	5.4	2.7	2.4	3.1	3.3
Serotransferrin	77.1	6.3	0.3	-0.19	-0.17	-0.23	-0.01	17.0	11.3	8.7	7.8	8.1
Serum Albumin	69.4	5.7	0.5	-0.19	0.11	0.24	0.00	27.5	12.7	15.1	15.9	15.8
Prothrombin	70.0	5.4	0.7	-0.17	-0.08	-0.04	0.29	1.0	0.6	0.8	0.6	0.5
Beta-actin-like Protein 2	42.0	5.2	0.3	0.09	-0.50	-0.25	0.08	8.4	11.1	9.4	9.3	8.2
Apolipoprotein A-I	30.8	5.4	1.7	0.11	-0.19	-0.06	0.25	2.9	3.1	3.3	3.4	2.9
Aplha-2-HS-Glycoprotein	39.3	5.2	0.5	0.15	-0.31	-0.24	-0.07	7.4	10.2	8.5	9.6	8.7
POTE Ankyrin Domain Family Member F	121.4	5.6	1.7	0.16	-0.16	-0.16	-0.13	1.9	3.3	2.8	2.5	3.0
Pregnancy Zone Protein	163.9	5.7	-3.4	0.17	-0.29	-0.24	0.14	0.5	0.8	0.8	0.7	0.6
Complement C3	187.2	5.8	-1.8	0.17	-0.24	-0.22	-0.07	1.6	2.4	2.0	2.3	2.1
Beta-2 Glycoprotein 1	38.3	7.1	0.1	0.17	0.27	0.22	-0.14	0.7	2.9	3.9	3.4	5.2
Tetranectin	22.5	5.4	0.2	0.17	0.28	0.26	-0.04	0.3	0.4	0.5	0.5	0.6
Ig Kappa Constant	11.8	5.8	0.7	0.17	0.27	0.24	0.11	0.2	0.3	0.4	0.4	0.4
Ig Lambda Constant 3	11.3	6.5	1.0	0.18	0.26	0.11	0.27	0.2	0.3	0.3	0.3	0.3
Hemoglobin Subunit Epsilon	16.2	8.0	0.1	0.19	-0.15	-0.19	0.19	0.8	3.5	3.4	2.9	2.6
Apolipoprotein C-III	10.9	5.1	0.9	0.19	0.06	0.11	0.27	0.3	0.8	1.2	1.0	0.9
Thrombospondin-1	129.4	4.6	1.1	0.19	0.18	0.26	0.01	0.2	0.9	1.2	1.1	1.3
Vitronectin	54.3	5.4	1.4	0.19	0.04	0.07	0.18	0.2	0.7	0.8	0.6	0.7
Alpha-2 Macroglobulin	163.3	5.8	-2.9	0.20	0.05	0.12	0.19	1.5	3.9	4.6	4.8	4.1
Inter-alpha-trypsin Inhibitor Heavy Chain H4	103.4	6.2	-0.7	0.20	-0.06	-0.23	0.10	0.7	3.0	2.8	2.9	2.6
Ig Heavy Constant Gamma-1	36.1	7.4	-0.1	0.20	0.10	0.23	0.14	0.3	0.5	0.6	0.6	0.6
Antithrombin-III	52.6	6.1	0.2	0.20	0.00	0.04	0.28	0.6	1.0	1.1	1.0	0.9
Hemoglobin Subunit Beta	16.0	6.5	0.2	0.20	-0.04	-0.01	0.30	0.1	0.6	0.7	0.7	0.5
Hemoglobin Subunit Delta	16.1	7.4	0.4	0.20	-0.04	-0.02	0.30	0.3	1.3	1.5	1.4	1.1
Actin, Cytoplasmic 2	41.8	5.2	0.4	0.20	-0.01	-0.02	0.03	0.1	0.5	0.5	0.6	0.5
Hemoglobin Subunit Alpha	15.3	8.1	0.5	0.20	0.06	0.23	-0.09	1.9	12.1	13.1	12.5	13.7
Gelsolin	85.7	5.7	0.7	0.20	0.09	0.18	0.22	0.2	0.5	0.7	0.6	0.6
Complement C9	63.2	5.3	0.8	0.20	-0.02	0.02	0.28	0.5	1.2	1.4	1.4	1.1

Figure S11. PC-1a sorted against protein MW, pI, I.O.S.S., PCs, and HC RPA.

	M.W.	pI	I.O.S.S.	PC-1a	PC-2a	PC-1b	PC-2b	Serum	GO	GO-N3	GO≡	C2GO
Beta-actin-like Protein 2	42.0	5.2	0.3	0.09	-0.50	-0.25	0.08	8.4	11.1	9.4	9.3	8.2
Aplha-2-HS-Glycoprotein	39.3	5.2	0.5	0.15	-0.31	-0.24	-0.07	7.4	10.2	8.5	9.6	8.7
Pregnancy Zone Protein	163.9	5.7	-3.4	0.17	-0.29	-0.24	0.14	0.5	0.8	0.8	0.7	0.6
Complement C3	187.2	5.8	-1.8	0.17	-0.24	-0.22	-0.07	1.6	2.4	2.0	2.3	2.1
Apolipoprotein A-I	30.8	5.4	1.7	0.11	-0.19	-0.06	0.25	2.9	3.1	3.3	3.4	2.9
Serotransferrin	77.1	6.3	0.3	-0.19	-0.17	-0.23	-0.01	17.0	11.3	8.7	7.8	8.1
POTE Ankyrin Domain Family Member F	121.4	5.6	1.7	0.16	-0.16	-0.16	-0.13	1.9	3.3	2.8	2.5	3.0
Hemoglobin Subunit Epsilon	16.2	8.0	0.1	0.19	-0.15	-0.19	0.19	0.8	3.5	3.4	2.9	2.6
Prothrombin	70.0	5.4	0.7	-0.17	-0.08	-0.04	0.29	1.0	0.6	0.8	0.6	0.5
Inter-alpha-trypsin Inhibitor Heavy Chain H4	103.4	6.2	-0.7	0.20	-0.06	-0.23	0.10	0.7	3.0	2.8	2.9	2.6
Hemoglobin Subunit Beta	16.0	6.5	0.2	0.20	-0.04	-0.01	0.30	0.1	0.6	0.7	0.7	0.5
Hemoglobin Subunit Delta	16.1	7.4	0.4	0.20	-0.04	-0.02	0.30	0.3	1.3	1.5	1.4	1.1
Complement C9	63.2	5.3	0.8	0.20	-0.02	0.02	0.28	0.5	1.2	1.4	1.4	1.1
Actin, Cytoplasmic 2	41.8	5.2	0.4	0.20	-0.01	-0.02	0.03	0.1	0.5	0.5	0.6	0.5
Antithrombin-III	52.6	6.1	0.2	0.20	0.00	0.04	0.28	0.6	1.0	1.1	1.0	0.9
Lactotransferrin	78.2	7.2	0.3	-0.20	0.01	0.18	0.22	2.7	0.8	0.9	0.9	0.9
Vitronectin	54.3	5.4	1.4	0.19	0.04	0.07	0.18	0.2	0.7	0.8	0.6	0.7
Alpha-2 Macroglobulin	163.3	5.8	-2.9	0.20	0.05	0.12	0.19	1.5	3.9	4.6	4.8	4.1
Apolipoprotein C-III	10.9	5.1	0.9	0.19	0.06	0.11	0.27	0.3	0.8	1.2	1.0	0.9
Hemoglobin Subunit Alpha	15.3	8.1	0.5	0.20	0.06	0.23	-0.09	1.9	12.1	13.1	12.5	13.7
Vitamin D Binding Protein	53.0	5.3	0.8	-0.20	0.07	0.09	-0.25	5.4	2.7	2.4	3.1	3.3
Inter-alpha-trypsin Inhibitor Heavy Chain H2	106.5	6.1	-1.1	-0.20	0.09	0.17	-0.23	13.9	4.9	5.0	5.9	7.0
Gelsolin	85.7	5.7	0.7	0.20	0.09	0.18	0.22	0.2	0.5	0.7	0.6	0.6
Ig Heavy Constant Gamma-1	36.1	7.4	-0.1	0.20	0.10	0.23	0.14	0.3	0.5	0.6	0.6	0.6
Serum Albumin	69.4	5.7	0.5	-0.19	0.11	0.24	0.00	27.5	12.7	15.1	15.9	15.8
Thrombospondin-1	129.4	4.6	1.1	0.19	0.18	0.26	0.01	0.2	0.9	1.2	1.1	1.3
Ig Lambda Constant 3	11.3	6.5	1.0	0.18	0.26	0.11	0.27	0.2	0.3	0.3	0.3	0.3
Beta-2 Glycoprotein 1	38.3	7.1	0.1	0.17	0.27	0.22	-0.14	0.7	2.9	3.9	3.4	5.2
Ig Kappa Constant	11.8	5.8	0.7	0.17	0.27	0.24	0.11	0.2	0.3	0.4	0.4	0.4
Tetranectin	22.5	5.4	0.2	0.17	0.28	0.26	-0.04	0.3	0.4	0.5	0.5	0.6

Figure S12. PC-2a sorted against protein MW, pI, I.O.S.S., PCs, and HC RPA

	M.W.	pI	I.O.S.S.	PC-1a	PC-2a	PC-1b	PC-2b	Serum	GO	GO-N3	GO≡	C2GO
Beta-actin-like Protein 2	42.0	5.2	0.3	0.09	-0.50	-0.25	0.08	8.4	11.1	9.4	9.3	8.2
Aplha-2-HS-Glycoprotein	39.3	5.2	0.5	0.15	-0.31	-0.24	-0.07	7.4	10.2	8.5	9.6	8.7
Pregnancy Zone Protein	163.9	5.7	-3.4	0.17	-0.29	-0.24	0.14	0.5	0.8	0.8	0.7	0.6
Serotransferrin	77.1	6.3	0.3	-0.19	-0.17	-0.23	-0.01	17.0	11.3	8.7	7.8	8.1
Inter-alpha-trypsin Inhibitor Heavy Chain H4	103.4	6.2	-0.7	0.20	-0.06	-0.23	0.10	0.7	3.0	2.8	2.9	2.6
Complement C3	187.2	5.8	-1.8	0.17	-0.24	-0.22	-0.07	1.6	2.4	2.0	2.3	2.1
Hemoglobin Subunit Epsilon	16.2	8.0	0.1	0.19	-0.15	-0.19	0.19	0.8	3.5	3.4	2.9	2.6
POTE Ankyrin Domain Family Member F	121.4	5.6	1.7	0.16	-0.16	-0.16	-0.13	1.9	3.3	2.8	2.5	3.0
Apolipoprotein A-I	30.8	5.4	1.7	0.11	-0.19	-0.06	0.25	2.9	3.1	3.3	3.4	2.9
Prothrombin	70.0	5.4	0.7	-0.17	-0.08	-0.04	0.29	1.0	0.6	0.8	0.6	0.5
Actin, Cytoplasmic 2	41.8	5.2	0.4	0.20	-0.01	-0.02	0.03	0.1	0.5	0.5	0.6	0.5
Hemoglobin Subunit Delta	16.1	7.4	0.4	0.20	-0.04	-0.02	0.30	0.3	1.3	1.5	1.4	1.1
Hemoglobin Subunit Beta	16.0	6.5	0.2	0.20	-0.04	-0.01	0.30	0.1	0.6	0.7	0.7	0.5
Complement C9	63.2	5.3	0.8	0.20	-0.02	0.02	0.28	0.5	1.2	1.4	1.4	1.1
Antithrombin-III	52.6	6.1	0.2	0.20	0.00	0.04	0.28	0.6	1.0	1.1	1.0	0.9
Vitronectin	54.3	5.4	1.4	0.19	0.04	0.07	0.18	0.2	0.7	0.8	0.6	0.7
Vitamin D Binding Protein	53.0	5.3	0.8	-0.20	0.07	0.09	-0.25	5.4	2.7	2.4	3.1	3.3
Apolipoprotein C-III	10.9	5.1	0.9	0.19	0.06	0.11	0.27	0.3	0.8	1.2	1.0	0.9
Ig Lambda Constant 3	11.3	6.5	1.0	0.18	0.26	0.11	0.27	0.2	0.3	0.3	0.3	0.3
Alpha-2 Macroglobulin	163.3	5.8	-2.9	0.20	0.05	0.12	0.19	1.5	3.9	4.6	4.8	4.1
Inter-alpha-trypsin Inhibitor Heavy Chain H2	106.5	6.1	-1.1	-0.20	0.09	0.17	-0.23	13.9	4.9	5.0	5.9	7.0
Lactotransferrin	78.2	7.2	0.3	-0.20	0.01	0.18	0.22	2.7	0.8	0.9	0.9	0.9
Gelsolin	85.7	5.7	0.7	0.20	0.09	0.18	0.22	0.2	0.5	0.7	0.6	0.6
Beta-2 Glycoprotein 1	38.3	7.1	0.1	0.17	0.27	0.22	-0.14	0.7	2.9	3.9	3.4	5.2
Hemoglobin Subunit Alpha	15.3	8.1	0.5	0.20	0.06	0.23	-0.09	1.9	12.1	13.1	12.5	13.7
Ig Heavy Constant Gamma-1	36.1	7.4	-0.1	0.20	0.10	0.23	0.14	0.3	0.5	0.6	0.6	0.6
Serum Albumin	69.4	5.7	0.5	-0.19	0.11	0.24	0.00	27.5	12.7	15.1	15.9	15.8
Ig Kappa Constant	11.8	5.8	0.7	0.17	0.27	0.24	0.11	0.2	0.3	0.4	0.4	0.4
Tetranectin	22.5	5.4	0.2	0.17	0.28	0.26	-0.04	0.3	0.4	0.5	0.5	0.6
Thrombospondin-1	129.4	4.6	1.1	0.19	0.18	0.26	0.01	0.2	0.9	1.2	1.1	1.3

Figure S13. PC-1b sorted against protein MW, pI, I.O.S.S., PCs, and HC RPA

	M.W.	pI	I.O.S.S.	PC-1a	PC-2a	PC-1b	PC-2b	Serum	GO	GO-N3	GO≡	C2GO
Vitamin D Binding Protein	53.0	5.3	0.8	-0.20	0.07	0.09	-0.25	5.4	2.7	2.4	3.1	3.3
Inter-alpha-trypsin Inhibitor Heavy Chain H2	106.5	6.1	-1.1	-0.20	0.09	0.17	-0.23	13.9	4.9	5.0	5.9	7.0
Beta-2 Glycoprotein 1	38.3	7.1	0.1	0.17	0.27	0.22	-0.14	0.7	2.9	3.9	3.4	5.2
POTE Ankyrin Domain Family Member F	121.4	5.6	1.7	0.16	-0.16	-0.16	-0.13	1.9	3.3	2.8	2.5	3.0
Hemoglobin Subunit Alpha	15.3	8.1	0.5	0.20	0.06	0.23	-0.09	1.9	12.1	13.1	12.5	13.7
Aplha-2-HS-Glycoprotein	39.3	5.2	0.5	0.15	-0.31	-0.24	-0.07	7.4	10.2	8.5	9.6	8.7
Complement C3	187.2	5.8	-1.8	0.17	-0.24	-0.22	-0.07	1.6	2.4	2.0	2.3	2.1
Tetranectin	22.5	5.4	0.2	0.17	0.28	0.26	-0.04	0.3	0.4	0.5	0.5	0.6
Serotransferrin	77.1	6.3	0.3	-0.19	-0.17	-0.23	-0.01	17.0	11.3	8.7	7.8	8.1
Serum Albumin	69.4	5.7	0.5	-0.19	0.11	0.24	0.00	27.5	12.7	15.1	15.9	15.8
Thrombospondin-1	129.4	4.6	1.1	0.19	0.18	0.26	0.01	0.2	0.9	1.2	1.1	1.3
Actin, Cytoplasmic 2	41.8	5.2	0.4	0.20	-0.01	-0.02	0.03	0.1	0.5	0.5	0.6	0.5
Beta-actin-like Protein 2	42.0	5.2	0.3	0.09	-0.50	-0.25	0.08	8.4	11.1	9.4	9.3	8.2
Inter-alpha-trypsin Inhibitor Heavy Chain H4	103.4	6.2	-0.7	0.20	-0.06	-0.23	0.10	0.7	3.0	2.8	2.9	2.6
Ig Kappa Constant	11.8	5.8	0.7	0.17	0.27	0.24	0.11	0.2	0.3	0.4	0.4	0.4
Pregnancy Zone Protein	163.9	5.7	-3.4	0.17	-0.29	-0.24	0.14	0.5	0.8	0.8	0.7	0.6
Ig Heavy Constant Gamma-1	36.1	7.4	-0.1	0.20	0.10	0.23	0.14	0.3	0.5	0.6	0.6	0.6
Vitronectin	54.3	5.4	1.4	0.19	0.04	0.07	0.18	0.2	0.7	0.8	0.6	0.7
Hemoglobin Subunit Epsilon	16.2	8.0	0.1	0.19	-0.15	-0.19	0.19	0.8	3.5	3.4	2.9	2.6
Alpha-2 Macroglobulin	163.3	5.8	-2.9	0.20	0.05	0.12	0.19	1.5	3.9	4.6	4.8	4.1
Lactotransferrin	78.2	7.2	0.3	-0.20	0.01	0.18	0.22	2.7	0.8	0.9	0.9	0.9
Gelsolin	85.7	5.7	0.7	0.20	0.09	0.18	0.22	0.2	0.5	0.7	0.6	0.6
Apolipoprotein A-I	30.8	5.4	1.7	0.11	-0.19	-0.06	0.25	2.9	3.1	3.3	3.4	2.9
Apolipoprotein C-III	10.9	5.1	0.9	0.19	0.06	0.11	0.27	0.3	0.8	1.2	1.0	0.9
Ig Lambda Constant 3	11.3	6.5	1.0	0.18	0.26	0.11	0.27	0.2	0.3	0.3	0.3	0.3
Complement C9	63.2	5.3	0.8	0.20	-0.02	0.02	0.28	0.5	1.2	1.4	1.4	1.1
Antithrombin-III	52.6	6.1	0.2	0.20	0.00	0.04	0.28	0.6	1.0	1.1	1.0	0.9
Prothrombin	70.0	5.4	0.7	-0.17	-0.08	-0.04	0.29	1.0	0.6	0.8	0.6	0.5
Hemoglobin Subunit Beta	16.0	6.5	0.2	0.20	-0.04	-0.01	0.30	0.1	0.6	0.7	0.7	0.5
Hemoglobin Subunit Delta	16.1	7.4	0.4	0.20	-0.04	-0.02	0.30	0.3	1.3	1.5	1.4	1.1

Figure S14. PC-2b sorted against protein MW, pI, I.O.S.S., PCs, and HC RPA

Table S1. Formulation for protein desorption buffer.

Component	Quantity	Final Conc.
0.5M Tris-HCL, pH 6.8	500 µL	62.5 mM
50% (v/v) Glycerol	15.00	25% (v/v)
SDS	80 mg	2% (w/v)
diH_2O	to 4 mL	

* Do not include any bromophenol blue.

**Always freshly prepared.

Table S2. Hard Corona Profiling by LC-MS

Hard Corona Proteins	Molecular Weight (kDa)	Isoelectric Point (pI)	Intrinsic Solubility Score^	Protein Functions	Relative Protein Abundance (%)				
					Control Serum	GO	GO-N_3	GO≡	C^2GO
Serum Albumin	69.37	5.67	0.52	Modulating blood osmotic pressure, a carrier for steroids, fatty acids, and thyroid hormones	27.46	12.72	15.13	15.89	[a]15.79
Serotransferrin (Transferrin)	77.06	6.29	0.27	Iron binding glycoprotein	17.02	11.31	8.72	7.75	8.13
Lactotransferrin (Lactoferrin)	78.18	7.23	0.30	Iron binding globular glycoprotein, innate immune system	2.70	0.81	0.91	0.86	0.85
Vitamin D Binding Protein (GC-Globulin)	52.96	5.27	0.76	Binds and transports Vitamin D	5.44	2.66	2.38	3.06	3.26
Prothrombin	70.04	5.44	0.75		0.98	0.64	0.75	0.62	0.53
Inter-alpha-trypsin Inhibitor Heavy Chain H2	106.46	6.10	-1.07	Bikunin (plasma proteinase inhibitor) binding	13.89	4.92	4.98	5.87	6.95
Inter-alpha-trypsin Inhibitor Heavy Chain H4	103.36	6.24	-0.74	Plasma Kallikrein-sensitive glycoprotein, dextran sulfate-binding	0.67	2.95	2.78	2.87	2.64
Gelsolin	85.70	5.67	0.73	ATP and lipid binding	0.17	0.51	0.67	0.58	0.57
Actin, Cytoplasmic 2	41.79	5.17	0.40	ATP binding	0.13	0.52	0.51	0.59	0.51
Beta-actin-like Protein 2	42.00	5.24	0.29	Various, e.g. ATP-binding and nucleotide-binding	8.44	11.06	9.37	9.34	8.20
Alpha-2 Macroglobulin	163.29	5.76	-2.91	Protease inhibitor	1.50	3.88	4.60	4.79	4.08
Pregnancy Zone Protein	163.86	5.72	-3.37	Protease inhibitor	0.49	0.83	0.75	0.71	0.63
POTE Ankyrin Domain Family Member F	121.44	5.59	1.67	Retina homeostasis	1.86	3.34	2.75	2.51	2.97
Aplha-2-HS-Glycoprotein	39.32	5.23	0.49	Promotes endocytosis, carrier for calcium and phosphate (e.g. calcium phosphate)	7.37	10.15	8.48	9.55	8.70
Apolipoprotein A-I	30.78	5.41	1.69	Major component of plasma HDL particle	2.91	3.14	3.30	3.36	2.85
Apolipoprotein C-III	10.85	5.11	0.86	Component of VLDL, inhibits hepatic uptake of triglyceride-rich particles	0.26	0.84	1.18	0.95	0.87
Hemoglobin Subunit Alpha	15.26	8.12	0.51	Oxygen, heme, and iron ion binding for oxygen transport	1.92	12.12	13.05	12.48	13.68
Hemoglobin Subunit Beta	16.00	6.49	0.17		0.11	0.62	0.75	0.71	0.63
Hemoglobin Submit Delta	16.06	7.42	0.40		0.28	1.26	1.49	1.35	1.08
Hemoglobin Subunit Epsilon	16.02	8.02	0.06		0.79	3.53	3.39	2.93	2.56
Ig Heavy Constant Gamma-1	36.11	7.38	-0.11	Antigen binding, immunoglobulin receptor binding	0.27	0.52	0.61	0.59	0.58
Ig Lambda Constant 3	11.27	6.53	0.95	Antigen binding	0.21	0.26	0.30	0.28	0.31
Ig Kappa Constant	11.77	5.85	0.73	Antigen binding, immunoglobulin receptor binding	0.19	0.27	0.42	0.40	0.39
Complement C3	187.15	5.77	-1.79	Contribute to innate immunity (compliment system)	1.64	2.40	2.02	2.32	2.08
Complement component C9	63.17	5.29	0.82	Contribute to innate immunity (compliment system)	0.48	1.18	1.39	1.36	1.08
Antithrombin-III	52.60	6.07	0.16	Serine protease inhibitor, inactivate coagulation, protease binding, *heparin binding.*	0.56	0.97	1.09	0.96	0.94
Thrombospondin-1	129.38	4.58	1.12	Glycoprotein that binds to reelin receptors, intact with cell adhesion receptors, and mediates cell-to-cell and cell-to-matrix interactions. *Binds heparin.*	0.19	0.88	1.21	1.10	1.26

Hard Corona Proteins	Molecular Weight (kDa)	Isoelectric Point (pI)	Intrinsic Solubility Score^	Protein Functions	Relative Protein Abundance (%)				
					Control Serum	GO	GO-N_3	GO≡	C^2GO
Beta-2 Glycoprotein 1	38.03	7.09	0.08	Known to interact with proteins/molecules with negative surface charges e.g. *heparin*, anionic phospholipids, and dextran sulfate.	0.69	2.89	3.87	3.44	5.23
Tetranectin	22.54	5.39	0.16	binds to plasminogen, *heparin binding*, Ca ion binding	0.32	0.44	0.54	0.52	0.58
Vitronectin	54.31	5.38	1.43	cell adhesion and spreading factor, collagen binding, extracellular matrix binding, *heparin binding*	0.17	0.66	0.83	0.57	0.67

^ Sequence-based intrinsic solubility score calculated by CamSol Intrinsic. <-1 = very hydrophobic, >1 = very hydrophilic.

Table S3.

Run#	Material	Factor A (PC-1b score)	Factor B (PC-2b score)	Factor C (Dose, µg/mL)	Response (Cell Viability, %)
1	GO	-5.42194	0.89008	10	55.05
2	GO	-5.42194	0.89008	10	55.54
3	GO	-5.42194	0.89008	10	60.08
4	GO	-5.42194	0.89008	10	64.03
5	$GO-N_3$	1.87868	4.11905	10	28.12
6	$GO-N_3$	1.87868	4.11905	10	29.02
7	$GO-N_3$	1.87868	4.11905	10	30.72
8	$GO-N_3$	1.87868	4.11905	10	33.31
9	GO≡	0.25004	0.51484	10	15.64
10	GO≡	0.25004	0.51484	10	15.64
11	GO≡	0.25004	0.51484	10	24.05
12	GO≡	0.25004	0.51484	10	35.06
13	C^2GO	3.29321	-3.78557	10	9.15
14	C^2GO	3.29321	-3.78557	10	5.76
15	C^2GO	3.29321	-3.78557	10	9.37
16	C^2GO	3.29321	-3.78557	10	5.08
17	GO	-5.42194	0.89008	50	48.50
18	GO	-5.42194	0.89008	50	53.53
19	GO	-5.42194	0.89008	50	59.45
20	GO	-5.42194	0.89008	50	65.83
21	$GO-N_3$	1.87868	4.11905	50	18.12
22	$GO-N_3$	1.87868	4.11905	50	25.29
23	$GO-N_3$	1.87868	4.11905	50	23.09
24	$GO-N_3$	1.87868	4.11905	50	22.02
25	GO≡	0.25004	0.51484	50	14.85
25	GO≡	0.25004	0.51484	50	17.62
27	GO≡	0.25004	0.51484	50	16.77
28	GO≡	0.25004	0.51484	50	19.14
29	C^2GO	3.29321	-3.78557	50	3.56
30	C^2GO	3.29321	-3.78557	50	3.78
31	C^2GO	3.29321	-3.78557	50	9.15
32	C^2GO	3.29321	-3.78557	50	5.82
33	GO	-5.42194	0.89008	100	24.90
34	GO	-5.42194	0.89008	100	21.29
35	GO	-5.42194	0.89008	100	24.56
36	GO	-5.42194	0.89008	100	17.45
37	$GO-N_3$	1.87868	4.11905	100	12.20
38	$GO-N_3$	1.87868	4.11905	100	14.34
39	$GO-N_3$	1.87868	4.11905	100	11.35
40	$GO-N_3$	1.87868	4.11905	100	12.65
41	GO≡	0.25004	0.51484	100	8.86
42	GO≡	0.25004	0.51484	100	8.92
43	GO≡	0.25004	0.51484	100	10.95
44	GO≡	0.25004	0.51484	100	8.70
45	C^2GO	3.29321	-3.78557	100	6.10
46	C^2GO	3.29321	-3.78557	100	3.56
47	C^2GO	3.29321	-3.78557	100	3.44
48	C^2GO	3.29321	-3.78557	100	3.78

Table S4. Sequential Model Sum of Squares (SMSS) for LDH Cell Viability (%)

Source	Sum of Squares	df*	Mean Square	F Value	*p*-value (Prob.>F)	
Mean vs Total	69.64	1	69.64			
Linear vs Mean	*6.10*	*3*	*2.03*	*190.29*	*<0.0001*	*Suggested*
2FI* vs Linear	0.055	3	0.018	1.79	0.1637	
Quadratic vs 2FI	0.042	1	0.042	4.48	0.0407	Aliased
Residual	0.37	40	0.009			
Total	76.21	48	1.59			

*df = degree of freedom, 2FI = two-factor interaction

Table S5. Lack of Fit Test for LDH Cell Viability (%)

Source	Sum of Squares	df	Mean Square	F Value	*p*-value* (Prob.>F)	
Linear	*0.015*	*8*	*0.019*	*2.11*	*0.0607*	*Suggested*
2FI	0.097	5	0.019	2.15	0.0821	
Quadratic	0.054	4	0.013	1.51	0.2207	Aliased
Pure Error	0.032	36	0.009			

*Models lack of fit should be insignificant ($p > 0.05$)

Table S6. Model Summary Statistic for LDH Cell Viability (%)

Source	Std. Dev.	R^2	Adjusted R^2	Predicted R^2	*PRESS**	
Linear	*0.10*	*0.9284*	*0.9236*	*0.9153*	*0.56*	*Suggested*
2FI	0.10	0.9367	0.9275	0.9168	0.55	
Quadratic	0.10	0.9431	0.9331	0.9208	0.52	Aliased

***PRESS:** predicted residual sum of squares

Table S7. Analysis of Variance Table for LDH Cell Viability (%)

Source	Sum of Squares	df*	Mean Square	F Value	*p*-value (Prob.>F)	
Model	6.10	3	2.03	190.29	<0.0001	*Significant*
Factor A (PC-1b score)	3.69	1	3.69	345.06	<0.0001	
Factor B (PC-2b score)	1.36	1	1.36	126.76	<0.0001	
Factor C (Dose, µg/mL)	1.06	1	1.06	99.03	<0.0001	
Residual	0.47	44	0.011			
Lack of Fit	0.15	8	0.019		0.0607	*Not Significant*
Pure Error	0.32	36	0.009			
Cor. Total	6.57	48				

Table S8. Model Coefficient Estimation

Factor	Estimated Coefficient	Df	Standard Error	95% CI Low	95% CI High	VIF
Intercept	1.30	1	0.016	1.27	1.33	
Factor A (PC-1b score)	-0.36	1	0.020	-0.40	-0.33	1.00
Factor B (PC-2b socre)	0.23	1	0.021	0.19	0.28	1.00
Factor C (Dose, µg/mL)	-0.18	1	0.018	-0.22	-0.14	1.00

References

[1] K.-C. Mei, Y. Guo, J. Bai, P. M. Costa, H. Kafa, A. Protti, R. C. Hider, K. T. Al-Jamal, ACS Applied Materials & Interfaces 2015, 7, 14176; K.-C. Mei, N. Rubio, P. M. Costa, H. Kafa, V. Abbate, F. Festy, S. S. Bansal, R. C. Hider, K. T. Al-Jamal, Chemical Communications 2015, 51, 14981.
[2] S. Schöttler, G. Becker, S. Winzen, T. Steinbach, K. Mohr, K. Landfester, V. Mailänder, F. R. Wurm, Nature Nanotechnology 2016, 11, 372.
[3] L. P. Kozlowski, Biology Direct 2016, 11, 55.
[4] P. Sormanni, F. A. Aprile, M. Vendruscolo, Journal of Molecular Biology 2015, 427, 478.
[5] H. Ali-Boucetta, K. T. Al-Jamal, K. Kostarelos, in *Biomedical Nanotechnology: Methods and Protocols*, (Ed: S. J. Hurst), Humana Press, Totowa, NJ 2011, 299.
[6] H. de Puig, I. Bosch, M. Carré-Camps, K. Hamad-Schifferli, Bioconjugate Chemistry 2017, 28, 230; W. Ma, A. Saccardo, D. Roccatano, D. Aboagye-Mensah, M. Alkaseem, M. Jewkes, F. Di Nezza, M. Baron, M. Soloviev, E. Ferrari, Nature Communications 2018, 9, 1489; F.-G. Natalia, B. Michaela, P. Sílvia, T. Sébastien, M. Tamoghna, B. Christian, L. Mika, A. Lorenzo, Small 2017, 13, 1701631; J. Kong, N. A. W. Bell, U. F. Keyser, Nano Letters 2016, 16, 3557.
